# Cross-Species Comparison between Mouse Kidney Multi-Omics and Human Genome-Wide Association Studies Highlights Molecular Features Associated with Oxalate Nephropathy

**DOI:** 10.34067/KID.0000001211

**Published:** 2026-06-11

**Authors:** Ryosuke Chaya, Kazumi Taguchi, Yutaka Hashimoto, Yuya Shirai, Ryuya Edahiro, Noah Sasa, Kenichi Yamamoto, Yoshihiro Tomofuji, Shinichi Namba, Kyuto Sonehara, Rei Unno, Shuzo Hamamoto, Atsushi Okada, John Muroski, Yoichi Kato, Ileana M. Cristea, Yukinori Okada, Takahiro Yasui

**Affiliations:** 1 Department of Nephro-Urology, Nagoya City University Graduate School of Medical Sciences, Nagoya, Japan; 2 Department of Statistical Genetics, Osaka University Graduate School of Medicine, Suita, Japan; 3 Department of Urology, University of Alabama at Birmingham, Birmingham, Alabama; 4 Department of Cell Biology, Nagoya City University Graduate School of Medical Sciences, Nagoya, Japan; 5 Department of Respiratory Medicine and Clinical Immunology, Osaka University Graduate School of Medicine, Suita, Japan; 6 Laboratory for Systems Genetics, RIKEN Center for Integrative Medical Sciences, Yokohama, Japan; 7 Department of Otorhinolaryngology-Head and Neck Surgery, Osaka University Graduate School of Medicine, Suita, Japan; 8 Department of Genome Informatics, Graduate School of Medicine, The University of Tokyo, Tokyo, Japan; 9 Division of Health Sciences, Laboratory of Children's Health and Genetics, Osaka University Graduate School of Medicine, Suita, Japan; 10 Department of Molecular Biology, Princeton University, Lewis Thomas Laboratory, Washington Road, Princeton, New Jersey; 11 Laboratory of Statistical Immunology, Immunology Frontier Research Center (WPI-IFReC), Osaka University, Suita, Japan; 12 Premium Research Institute for Human Metaverse Medicine (WPI-PRIMe), Osaka University, Suita, Japan

**Keywords:** gene expression, human genetics, kidney stones, proteomics

## Abstract

**Key Points:**

- The cross-species integration of mouse kidney multi-omics and human genome-wide association study identified 46 cross-species urolithiasis-related molecules.
- Cell type enrichment suggested that immune cells and collecting duct principal cells may play roles in urolithiasis pathophysiology.
- Follow-up analyses using human renal data, colocalization, and Mendelian randomization supported the relevance of the detected 46 molecules in humans.

**Background:**

Urolithiasis, a recurrent and increasingly prevalent disease worldwide, has a complex pathophysiology that hinders a comprehensive understanding.

**Methods:**

We performed a cross-species integrative analysis combining multi-omics profiling of kidneys from a hyperoxaluric mouse model and summary statistics from a large-scale human genome-wide association study (GWAS).

**Results:**

In the multi-omics analysis of kidneys from a hyperoxaluric mouse model using RNA-seq, whole-cell proteomics, and phosphoproteomics, we identified 1173 genes, 342 proteins, and 516 phosphorylated peptides that were differentially expressed compared with control mice. We used publicly available large-scale meta-GWAS summary statistics of urolithiasis from BioBank Japan, UK Biobank, and FinnGen (*n*=198,769) and prioritized genes using gene enrichment analysis. Through cross-species integration of mouse and human omics, we identified 46 molecules potentially relevant to urolithiasis, hereafter referred to as cross-species urolithiasis-related molecules. We examined the expression and genetic associations of these 46 molecules in human urolithiasis. Among these, *CRYAB* and *SHROOM3* showed differential expression in human renal papilla tissues with and without stones. Colocalization analysis between urolithiasis GWAS and expression quantitative trait loci data of human renal tubules and glomeruli suggested shared signals at the *GLUD1*, *UMOD*, *SLC34A1*, *TCEA3*, and *H1-0* regions. Mendelian randomization analysis using the same renal expression quantitative trait loci datasets further supported these findings, with *GLUD1*, *UMOD*, *TCEA3*, and *H1-0* showing statistically significant associations, suggesting a strong genetic link with urolithiasis.

**Conclusions:**

The study's findings highlight a set of cross-species urolithiasis-related molecules, supported by both expression and genetic evidence, offering insights for future functional validation and mechanistic investigations.

## Visual Abstract

**Figure:**
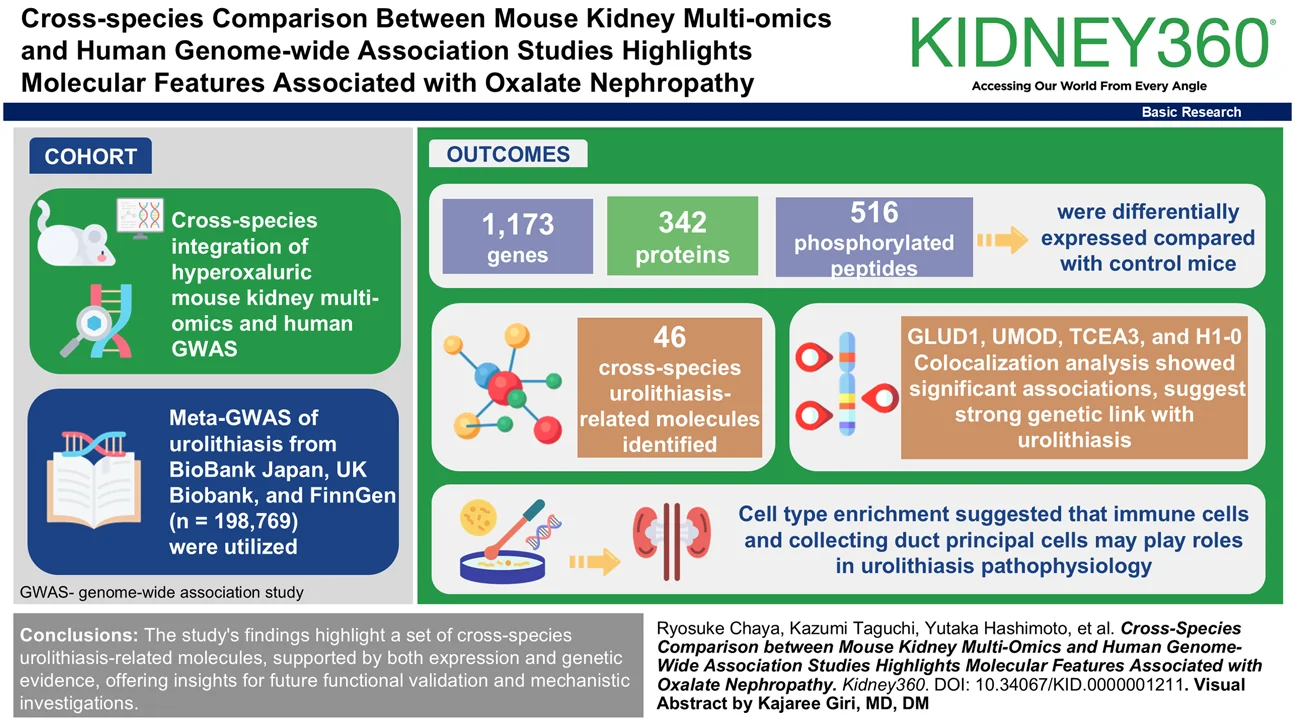

## Introduction

Urolithiasis is a condition that has a significant effect on the quality of life because of its severe pain, and it also poses a risk to life expectancy through the potential for urinary tract infections and irreversible kidney injury. According to recent epidemiologic studies, the lifetime prevalence of urolithiasis is estimated to be 10%–15% in men and 7% in women in Europe, the United States, and Japan.^1–4^ In addition, urolithiasis is a persistent disease characterized by a high recurrence rate, with up to 50% of patients experiencing recurrence within a decade.^5,6^ The pathophysiology of urolithiasis is complex, influenced by both genetic and acquired factors.^7^

Genome-wide association study (GWAS) has been used as a genetic approach to elucidate the pathogenesis of urolithiasis. According to twin studies, the heritability of urolithiasis is estimated to be 57% in men and 46% in women. This high heritability underscores the importance of approaching urolithiasis research from a genetic perspective.^8,9^ In a recent large-scale urolithiasis GWAS, Tanikawa *et al.* identified 14 significant loci, including nine novel loci, in a urolithiasis GWAS involving 198,769 individuals (11,130 cases and 187,639 controls).^10^ Urolithiasis has occasionally been included as one of the target traits in a phenome-wide association study (PheWAS). In this study, we used data from a large-scale PheWAS performed by Sakaue *et al.* using BioBank Japan (BBJ), which included urolithiasis as one of the analyzed phenotypes.^11^

Another way to investigate the pathogenesis of urolithiasis is molecular biologic approaches, such as animal models and human specimens. Our research team developed mouse models of hyperoxaluric nephrocalcinosis in the kidney and identified molecules associated with urolithiasis.^12–15^ Gene expression in human renal papilla specimens, the site of urinary stone formation, revealed that *FABP4* is associated with urolithiasis.^16^ GWAS and molecular biology approaches have advanced urolithiasis research but remain limited in identifying causal molecules and distinguishing cause from consequence, highlighting the need for integrative multi-omics and cross-species analyses.

In this research, we conducted a cross-species multi-omics analysis combining kidneys from a hyperoxaluric mouse model and urolithiasis GWAS data. We aimed to prioritize candidate molecules that may play functional roles in the mouse kidney and look for genetic associations in human GWAS data. The relevance of these candidate molecules was further assessed using additional urolithiasis-associated datasets. Analyzing the transcriptome, whole-cell (WC) proteome, and phosphoproteome in the kidneys of the hyperoxaluric nephrocalcinosis mouse model identified potential links to urolithiasis. GWAS summary statistics for urolithiasis from large-scale trans-ethnic meta-GWAS from BBJ, UK Biobank, and FinnGen (*n*_Total_=198,769) were used for gene enrichment analysis using Multi-marker Analysis of GenoMic Annotation (MAGMA).^17^ Incorporating data from mouse models and human studies, our study prioritized cross-species urolithiasis-related molecules. Using publicly available single-cell RNA-seq data, we explored the cell types from which the molecules identified in the above analyses originated. Finally, we assessed their potential involvement in human urolithiasis using gene-expression data from human renal papillae of patients with urolithiasis and colocalization and Mendelian randomization (MR) analyses between GWAS and kidney expression quantitative trait loci (eQTL) data.

## Methods

### Animal Procedures

A hyperoxaluric mouse model was generated by intraperitoneal injection of glyoxylate (100 mg/kg).^12^ Male C57BL/6J mice (8–10 week old) were divided into five groups (*n*=4): control, day 1, day 3, day 6 (1, 3, or 6 days of treatment), and day 12 (6-day treatment plus 6-day washout).

### Histology

The kidneys were fixed in 4% formalin, dehydrated, and embedded in paraffin for histologic analysis. Evaluation of calcium oxalate crystals within the kidneys was conducted using Pizzolato staining.

### Protein Quantification Using Mass Spectrometry Analysis

Proteins from mouse kidney tissues were precipitated with methanol–chloroform. The extracted proteins were digested with trypsin and analyzed as peptides for WC proteomics and phosphoproteomics. For phosphoproteomic analysis, phosphopeptides were enriched with TiO_2_ resin and used for subsequent quantification. Peptides were analyzed by nanoflow liquid chromatography-tandem mass spectrometry.

### Multi-Omics Analysis of Murine Kidney RNA-Seq, WC Proteome, and Phosphoproteome

To evaluate molecular changes quantified by RNA-seq and proteomics analyses, differential expression analysis was performed across three time points (day 1, day 3, and day 6) in the model mouse kidney. To characterize the biologic functions highlighted by these differentially expressed molecules, gene set enrichment analysis was performed using gene ontology (GO) Biological Process annotations. Furthermore, for phosphoproteome data, kinase set enrichment analysis was used to identify upstream kinases that may be responsible for the observed phosphorylation changes, using kinase-substrate relationship data from PhosphoSitePlus.^18^ Differentially expressed genes, proteins, and peptides were defined as those with a false discovery rate (FDR) <0.05.

### GWAS Summary Analysis

We analyzed publicly available GWAS summary data on urolithiasis to quantify human genetic risk at the molecular and cell type levels. The GWAS summary used in this study was a meta-analysis of urolithiasis GWAS results from a Japanese cohort (BBJ) and European cohorts (UK Biobank and FinnGen) and corresponds to the data used in the PheWAS study by Sakaue *et al.*^11^ To identify genes associated with urolithiasis, we performed gene enrichment analysis using MAGMA.^17^ Genes were considered significant at an FDR <0.05 in MAGMA analyses. To assess the enrichment of genetic disease risk across organs identified by GWAS, we use FUnctional Mapping and Annotation of Genetic Association (FUMA)^19^ and partitioned linkage disequilibrium score regression (partitioned LDSC).^20,21^ To investigate which biologic functions are enriched in the human urolithiasis GWAS, we performed gene set enrichment analysis using MAGMA based on GO biological process annotations. To evaluate cell type enrichment, we employed both linkage disequilibrium score regression specifically expressed genes^22^ and single-cell disease relevance score (scDRS)^23^ using publicly available single-cell RNA-seq data from mouse kidneys^24^ and human kidneys as references. The mouse dataset was obtained from the study conducted by Miao *et al.*, and the human dataset was derived from the Kidney Precision Medicine Project (KPMP).

### Integration of Human and Mouse Data

Human gene IDs were converted to mouse gene IDs using an ortholog reference to integrate human and mouse data. Ortholog reference data were obtained from Mouse Gene Informatics.^25^ The number of concurrently significant molecules or gene sets in both analyses was assessed using the Fisher exact test. The significance threshold for this assessment was set at an FDR <0.05. We define molecules that are significant (FDR <0.05) in both mouse and human data as cross-species urolithiasis-related molecules.

### Cell Type Deconvolution Analysis

Cell type deconvolution of bulk RNA-seq data from the hyperoxaluric mouse model was performed using CIBERSORTx^26^ to evaluate whether glyoxylate administration altered cellular composition and to assess the impact of these changes on transcriptomic profiles. We used a mouse single-cell RNA-seq dataset, the same one used in scDRS analysis, as the reference.

### Renal Papillae Microarray Analysis

Gene expression analysis of renal papillae, the site of stone formation in patients with urolithiasis, was conducted using microarray data from the Gene Expression Omnibus (GEO) repository (GSE73680).^27^ From this dataset, we extracted data from patients with urolithiasis only and performed differential expression analysis between renal papillae with Randall plaque and those without.

### Colocalization Analysis

We conducted a colocalization analysis to assess whether effective variants shared between urolithiasis GWAS and eQTL summary statistics were present within the gene loci corresponding to cross-species urolithiasis-related molecules. In this study, we refer to single-nucleotide polymorphisms as variants. The coloc.abf function in the R package coloc^28^ was used for analysis, computing the posterior probabilities of four hypotheses: H1 (no GWAS showing effects), H2 (effects present in one GWAS only), H3 (effects present in both GWAS without colocalization), and H4 (effects present in both GWAS with colocalization).^28^ An H4 posterior probability >75% was defined as indicative of colocalization.

### MR Analysis

To investigate whether kidney eQTLs exert a causal effect on urolithiasis, we performed MR analysis on the variants of genes corresponding to cross-species urolithiasis-related molecules. The analysis was conducted using the TwoSampleMR package^29^ in R. Variants with *P* ≤ 5.0×10^−8^ in the eQTL dataset were selected as instrumental variables.

### Statistical Analysis

The Benjamini–Hochberg method was used for multiple testing correction, with a significance level set at FDR <0.05.

Additional details on methods are provided in the Supplemental Methods.

## Results

### Three Modalities of Multi-Omics Analysis of the Kidney in a Hyperoxaluric Mouse Model

To comprehensively capture molecules functioning in the kidneys where calcium oxalate stones are formed, we conducted a multi-omics analysis using RNA-seq, WC proteome, and phosphoproteome on the kidneys of the hyperoxaluric mouse model. We confirmed the presence of calcium oxalate crystals in the kidneys (Figure 1A). Blood and urine biochemical test results are also presented in Supplemental Table 1. These results confirmed that the hyperoxaluric mouse model was successfully established.

**Figure 1:**
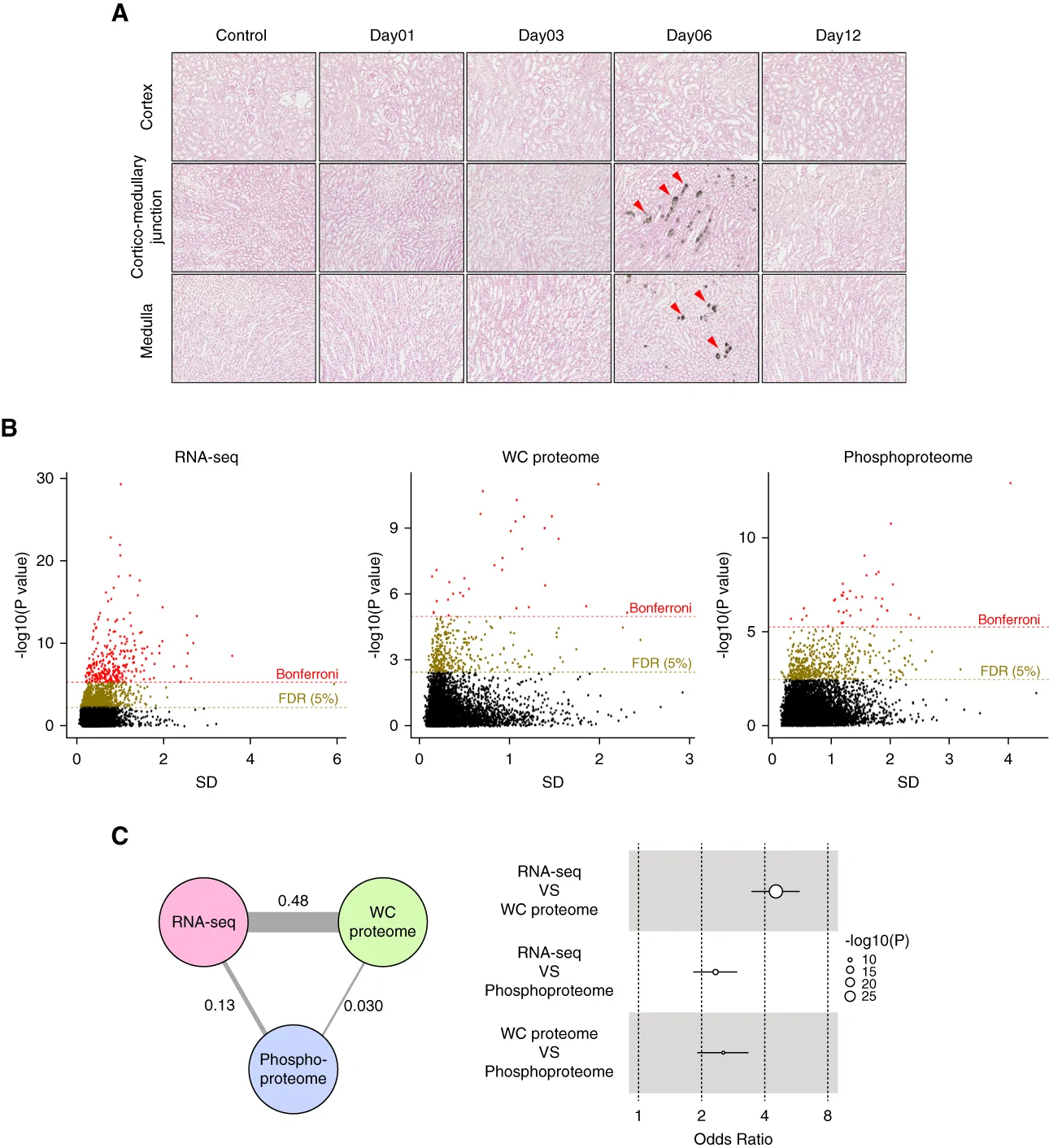
**Multi-omics analysis of the kidney from a hyperoxaluric mouse using the three modalities.** (A) The presence of calcium oxalate crystals in the kidneys of mice was confirmed by Pizzolato staining. In the images of the corticomedullary junction and medulla, the tubular lumen of the renal tubules and loop of Henle was visible throughout most regions. Calcium oxalate crystals were found in the tubular lumen of the medullary and corticomedullary junction and medulla in the glyoxylate 6-day group (the day 6 group). (B) Scatter plot of variability and significance depicting the differentially expressed molecules in RNA-seq (left), WC proteome (middle), and phosphoproteome (right), with the *x* axis representing the SD and the *y* axis representing the negative logarithm of the *P* value. The plots highlight the differentially expressed molecules. (C) Left: the correlation coefficients for the expression profiles across each modality are illustrated. Right: Fisher exact test was conducted to assess the degree of overlap between differentially expressed molecules for each modality. FDR, false discovery rate; WC, whole-cell.

After quantifying RNA, proteins, and phosphorylated peptides extracted from the kidneys of this mouse model, we conducted differential expression analysis. The analyses of variance demonstrated that 1173 of 10,828, 342 of 6969, and 516 of 7217 molecules were significantly differentially expressed in the RNA-seq, WC proteome, and phosphoproteome, respectively (Figure 1B). The expression profiles measured for each modality are shown in Supplemental Figure 1A. We analyzed the quantitative connections between molecules quantified by these three methods. The proteomic quantification of proteins and phosphorylated peptides involves annotating them with the original genes before transcription (see Supplemental Methods: proteomics data analysis). Molecules quantified across three modalities are shown in Supplemental Figure 1B, and the normalized expression levels for each pair of modalities are presented as scatter plots in Supplemental Figure 1C. The Pearson correlation coefficients were 0.48 between RNA-seq and the WC proteome, 0.13 between RNA-seq and the phosphoproteome, and 0.03 between the WC proteome and the phosphoproteome (Figure 1C). In Supplemental Figure 1D, the correlation was also assessed using linear regression. We also evaluated the shared differentially expressed molecules between the two modalities using the Fisher exact test. The odds ratios (ORs) were 4.4 for RNA-seq versus WC proteome, 2.3 for RNA-seq versus phosphoproteome, and 2.4 for WC proteome versus phosphoproteome, with corresponding *P* = 9.7×10^−25^, 6.9×10^−11^, and 7.0×10^−9^, respectively (Figure 1C). The observed trend of a higher correlation between RNA-seq and the WC proteome, alongside a lower correlation between the phosphoproteome and other modalities, as seen in our analysis of mouse kidneys, is consistent with previous reports. The low correlation between the phosphoproteome and the other modalities suggests that the phosphoproteome captures distinct biologic processes.

Subsequently, a gene set enrichment analysis using GO biological process was conducted to ascertain the functional roles of the molecules identified through differential expression analysis.^30^ The RNA-seq enrichment was mainly associated with inflammatory and injury-related processes along with metabolic processes in monocarboxylic acid and reactive oxygen species. In contrast, WC proteome enrichment predominantly reflected cellular energy and lipid metabolism among organic/oxoacid, fatty acid oxidation, lipid catabolism, and purine- and ATP-related pathways (Supplemental Figure 2, A and B). To further characterize the phosphopeptides identified in the phosphoproteome, we performed kinase set enrichment analysis to determine which downstream phosphorylation targets were altered (Supplemental Figure 2C). This analysis revealed enhanced phosphorylation downstream of mammalian target of rapamycin (mTOR; OR=3.8, *P* = 0.0047). Previous studies have shown that mTOR can promote nephrocalcinosis by inhibiting autophagy in renal tubules.^31^ However, mTOR does not emerge as a significant locus in the subsequent GWAS analyses.

### Gene-, Pathway-, and Organ-Level Analysis of Urolithiasis GWAS Summary Statistics

We analyzed publicly available GWAS summary statistics to elucidate the genetic background of urolithiasis. GWAS summary statistics for urolithiasis were obtained through a meta-analysis of Japanese GWAS (BBJ) and European GWAS (UK BioBank and FinnGen).^32–34^ In the BBJ GWAS and European GWAS, there were 20 and ten loci, respectively (Supplemental Figure 3A). Owing to the increased statistical power of cross-population meta-analysis, 33 loci were identified (Figure 2A).

**Figure 2:**
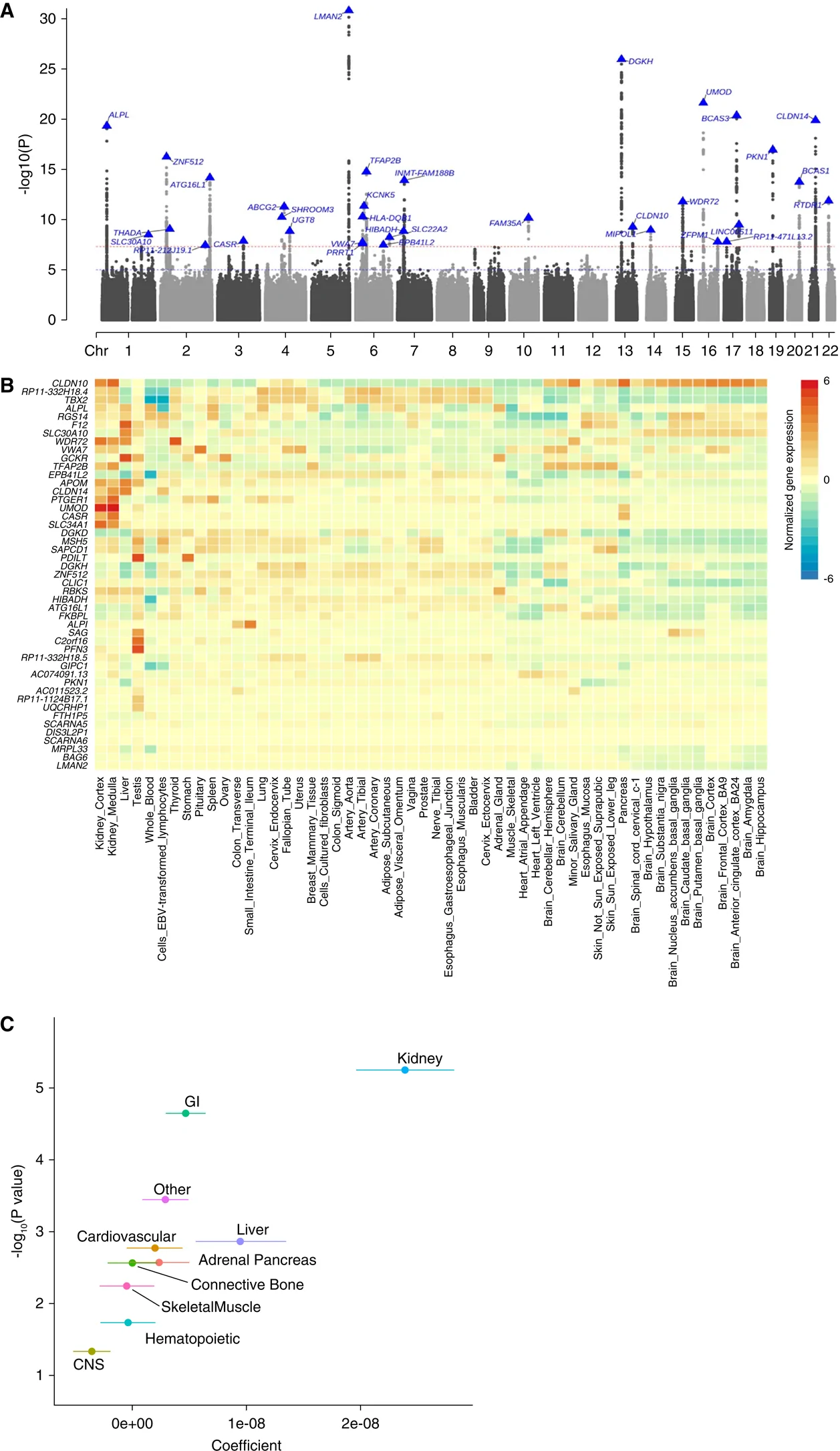
**Tissue-specific and kidney-enriched genetic architecture of urolithiasis revealed by GWAS summary statistics.** (A) The Manhattan plot derived from GWAS findings of urolithiasis exhibits a visual representation of the results. META data were obtained by combining Japanese GWAS (BBJ) and EUR (UK Biobank and FinnGen). The blue dashed line represents *P* value < 1×10^−5^, while the red dashed line indicates *P* value < 5×10^−8^. (B) Assessment of the expression of the top 50 genes with low *P* values, as identified using MAGMA and FUMA, across specific tissues. The colors in the heatmap represent the normalized gene expression levels by gene (by row), with an average value of zero. The gene expression data were obtained from GTEx Analysis Release V8. (C) We used partitioned LDSC to identify which organ-related regions on the chromosome were enriched with heritability. The heritability of urolithiasis GWAS is predominantly enriched in the kidney-related areas. BBJ, BioBank Japan; CNS, central nervous system; EUR, European genome-wide association study; FUMA, FUnctional Mapping and Annotation of Genetic Association; GI, gastrointestinal; GTEx, genotype-tissue expression; GWAS, genome-wide association study; MAGMA, Multi-marker Analysis of GenoMic Annotation; META, meta-analysis; partitioned LDSC, partitioned linkage disequilibrium score regression.

In the gene enrichment analysis, MAGMA^17^ evaluated *P* values for 40,649 genes, of which 343 were identified as significantly associated genes. Regarding gene set–level associations, we performed MAGMA-based gene set enrichment analysis using GO biological process annotations to identify biologic functions enriched in the urolithiasis GWAS (Supplemental Figure 3B). This analysis identified 97 GO terms that were significantly associated with urolithiasis after multiple testing correction. Examination of the top-ranked terms revealed prominent enrichment for immune-related processes as well as for signaling pathways such as phospholipase C/protein kinase C signaling. In addition, renal urate salt excretion and renal system processes were also enriched, suggesting that both immune regulation and kidney-specific biologic functions are genetically associated with urolithiasis.

To assess which organs showed enrichment of genetic risk for urolithiasis, we performed FUMA^19^ and partitioned LDSC^20^ analyses. Using the GENE2FUNC function in FUMA, we evaluated the top 50 genes identified by gene enrichment analysis (Figure 2B).^1^ The results showed that many kidney-specific genes were among the enriched genes. In the partitioned LDSC results, the genetic risk was also enriched in kidney-associated regions (Figure 2C). These results suggest that urolithiasis is a disease with a strong genetic connection to the kidneys.

### Cross-Species Identification of Urolithiasis-Associated Molecules and Biologic Processes

Building on the mouse multi-omics analyses and the human GWAS results presented above, we next performed a cross-species integrative analysis to identify shared urolithiasis-associated molecules and biologic processes at both the gene and gene set levels. We conducted an analysis to identify biologic similarities between the kidney multi-omics results from mouse models and the GWAS results for urolithiasis. A total of 46 molecules, including 31 genes identified through RNA-seq, two proteins from the WC proteome, and 21 peptides from the phosphoproteome, were found to be significantly differentially expressed in mice and were identified as human significant genes from gene enrichment analysis with MAGMA (Table 1 and Supplemental Table 2). We define these molecules as cross-species urolithiasis-related molecules. To confirm that the identification of these 46 molecules was not incidental, we demonstrated that genes with low *P* values in both human and mouse data were significantly enriched. Specifically, enrichment was evaluated by comparing the observed overlap with that expected under a null model in which genes were randomly distributed across datasets using the Fisher exact test. A significant overlap between the molecules identified by human MAGMA analysis and those differentially expressed in mouse analysis was observed in the RNA-seq (OR=1.9, *P* = 2.5×10^−3^) and phosphoproteome (OR=1.8, *P* = 0.04) datasets (Figure 3A). WC proteome analysis showed no significant overlap with human data (OR=0.27, *P* = 0.04).

**Table 1:** Cross-species urolithiasis-associated molecules obtained from a human genome-wide association study and mouse kidney multi-omics analysis

Human Gene Symbol	GWAS Significant Population	Previously Reported	Phosphorylation Site Position	Human MAGMA *P* Value	Mouse RNA-Seq *P* Value	Mouse WC Proteome *P* Value	Mouse Phosphoproteome *P* Value	Significant Modality	Specific Cell Type
*ABCG2*	BBJ, META	Reported	Unknown	2.8×10^−4a^	0.11	0.12	7.3×10^−4a^	Phospho	Proximal
*APOM*	Nothing	Not reported	NA	7.4×10^−8a^	1.4×10^−3a^	NA	NA	RNA-seq	Proximal
*BAG6*	Nothing	Not reported	T985, S986, S995	1.5×10^−7a^	0.31	0.43	5.3×10^−3a^	Phospho	Not specific
*C4A*	Nothing	Not reported	NA	4.5×10^−5a^	1.2×10^−7a^	NA	NA	RNA-seq	Proximal
*CASR*	META	Reported	S1062	2.6×10^−8a^	0.63	0.31	1.7×10^−4a^	Phospho	Not specific
*CDK12*	Nothing	Not reported	S331, S332, S333	3.4×10^−7a^	0.65	0.35	5.2×10^−4a^	Phospho	Not specific
			S337						
*CLDN10*	META	Not reported	NA	7.2×10^−8a^	4.6×10^−3a^	NA	NA	RNA-seq	Not specific
*CRYAB*	Nothing	Not reported	NA	2.5×10^−4a^	7.8×10^−4a^	NA	NA	RNA-seq	PC
*DDAH2*	Nothing	Not reported	NA	2.4×10^−6a^	8.6×10^−4a^	NA	NA	RNA-seq	Not specific
*EPB41L3*	Nothing	Not reported	S451, S456, S458	1.1×10^−5a^	0.38	0.19	4.8×10^−4a^	Phospho	Not specific
			Y464						
*FADS2*	Nothing	Not reported	NA	1.8×10^−5a^	1.4×10^−3a^	NA	NA	RNA-seq	Proximal
*FBN1*	Nothing	Not reported	NA	3.5×10^−4a^	7.4×10^−10a^	NA	NA	RNA-seq	Stroma
*GLUD1*	BBJ, META	Not reported	T409	1.5×10^−5a^	0.66	0.01	4.1×10^−3a^	Phospho	Not specific
			T410						
*H1-0*	Nothing	Not reported	NA	3.2×10^−5a^	1.5×10^−3a^	0.15	0.16	RNA-seq	Not specific
*HIBADH*	META	Not reported	S87	2.9×10^−9a^	3.1×10^−3a^	0.17	1.1×10^−3a^	RNA-seq	Not specific
								Phospho	
*HLA-B*	Nothing	Not reported	NA	2.7×10^−6a^	8.9×10^−7a^	NA	NA	RNA-seq	Not specific
*HLA-DQA1*	EUR	Not reported	NA	6.3×10^−5a^	4.5×10^−7a^	NA	NA	RNA-seq	Immune
*HLA-DRB1*	Nothing	Not reported	NA	7.1×10^−5a^	1.1×10^−8a^	NA	NA	RNA-seq	Immune
*HMGCS1*	Nothing	Reported	NA	1.6×10^−4a^	1.2×10^−4a^	NA	NA	RNA-seq	Not specific
*HSPA12A*	Nothing	Not reported	NA	5.0×10^−4a^	9.7×10^−4a^	NA	NA	RNA-seq	Not specific
*HSPA1B*	Nothing	Not reported	T222	2.7×10^−5a^	1.6×10^−5a^	0.53	9.0×10^−4a^	RNA-seq	Not specific
			T226		3.2×10^−5a^			Phospho	
*LUZP1*	Nothing	Not reported	S261	7.1×10^−4a^	0.31	0.75	9.3×10^−3a^	Phospho	Not specific
*MAPT*	Nothing	Not reported	S502, T504, S506	6.2×10^−4a^	5.6×10^−5a^	0.06	3.3×10^−3a^	RNA-seq	Proximal
			T509, T512					Phospho	
*MXD3*	BBJ, EUR, META	Not reported	NA	1.8×10^−4a^	3.3×10^−3a^	NA	NA	RNA-seq	Immune
*NCCRP1*	Nothing	Not reported	S40, T42, S43	6.3×10^−5a^	4.2×10^−8a^	0.12	1.3×10^−3a^	RNA-seq	Distal
			T46					Phospho	
*NDUFAB1*	Nothing	Not reported	NA	5.4×10^−4a^	1.5×10^−3a^	NA	NA	RNA-seq	Not specific
*PLCB1*	Nothing	Reported	NA	4.4×10^−4a^	2.6×10^−4a^	NA	NA	RNA-seq	Proximal
*PLCL1*	Nothing	Not reported	T94, S96	1.5×10^−4a^	0.44	0.27	1.0×10^−2a^	Phospho	Not specific
*PPHLN1*	Nothing	Not reported	S135	5.7×10^−4a^	0.81	0.88	8.9×10^−3a^	Phospho	Not specific
*PRRC2A*	Nothing	Not reported	T377, S378	3.3×10^−7a^	0.94	0.06	7.6×10^−3a^	Phospho	Not specific
*RAP1GAP*	BBJ, EUR, META	Not reported	NA	1.0×10^−6a^	0.36	1.6×10^−4a^	0.02	WC	Not specific
*SHROOM3*	META	Not reported	S888, T889	9.2×10^−6a^	0.31	NA	9.1×10^−4a^	Phospho	PC
*SLC12A3*	Nothing	Reported	T122, S124	7.2×10^−5a^	0.04	0.03	8.4×10^−4a^	Phospho	Distal
*SLC20A1*	Nothing	Not reported	NA	7.9×10^−4a^	4.5×10^−4a^	NA	NA	RNA-seq	Not specific
*SLC25A21*	META	Not reported	NA	6.0×10^−4a^	2.4×10^−4a^	NA	NA	RNA-seq	Proximal
*SLC34A1*	BBJ, EUR, META	Reported	NA	5.0×10^−10a^	3.5×10^−4a^	NA	NA	RNA-seq	Proximal
*SLCO4C1*	Nothing	Not reported	S24, S26, S28	5.9×10^−4a^	1.6×10^−4a^	0.72	5.0×10^−4a^	RNA-seq	PC
								Phospho	
*SNX17*	Nothing	Not reported	S434, S437, S440	4.3×10^−4a^	0.33	0.94	3.8×10^−3a^	Phospho	Not specific
*STAB1*	Nothing	Not reported	NA	2.4×10^−4a^	1.6×10^−3a^	NA	NA	RNA-seq	Immune
*TCEA3*	Nothing	Not reported	NA	5.9×10^−5a^	2.6×10^−3a^	NA	NA	RNA-seq	Proximal
*TMEM252*	Nothing	Not reported	NA	3.3×10^−5a^	1.1×10^−7a^	NA	NA	RNA-seq	Proximal
*TRPM7*	Nothing	Not reported	S1300	1.3×10^−4a^	0.38	0.95	1.1×10^−3a^	Phospho	Not specific
*TRPS1*	Nothing	Not reported	NA	3.4×10^−4a^	3.7×10^−3a^	NA	NA	RNA-seq	Not specific
*UGT8*	EUR, META	Not reported	NA	2.8×10^−5a^	8.4×10^−4a^	NA	NA	RNA-seq	Not specific
*UMOD*	BBJ, EUR, META	Reported	Unknown	3.4×10^−10a^	0.01	4.4×10^−5a^	6.3×10^−3a^	WC	Distal
								Phospho	
*VCL*	Nothing	Not reported	S795	5.1×10^−4^	2.2×10^−3a^	0.32	3.3×10^−8a^	RNA-seq	Not specific
								Phospho	

The column GWAS Significant Population indicates the population in which genome-wide significant variants were identified at the corresponding gene locus: the Japanese population (BioBank Japan), the European population, or a meta-analysis of Japanese and European populations.

The column Significant Modality denotes the modality or modalities in which the gene was found to be significant in the mouse multi-omics analyses.

Not specific means that they were not expressed specifically in a particular cell type in the mouse kidney single-cell RNA-seq data. BBJ, BioBank Japan; Distal, distal tubular cells; EUR, European genome-wide association study; GWAS, genome-wide association study; IC, intercalated cell; Immune, immune cells; MAGMA, Multi-marker Analysis of GenoMic Annotation; META, meta-analysis; NA, not available; PC, principal cell; Phospho, phosphoproteome; Proximal, proximal tubular cells; Stroma, stromal cells; WC, whole-cell.

**Figure 3:**
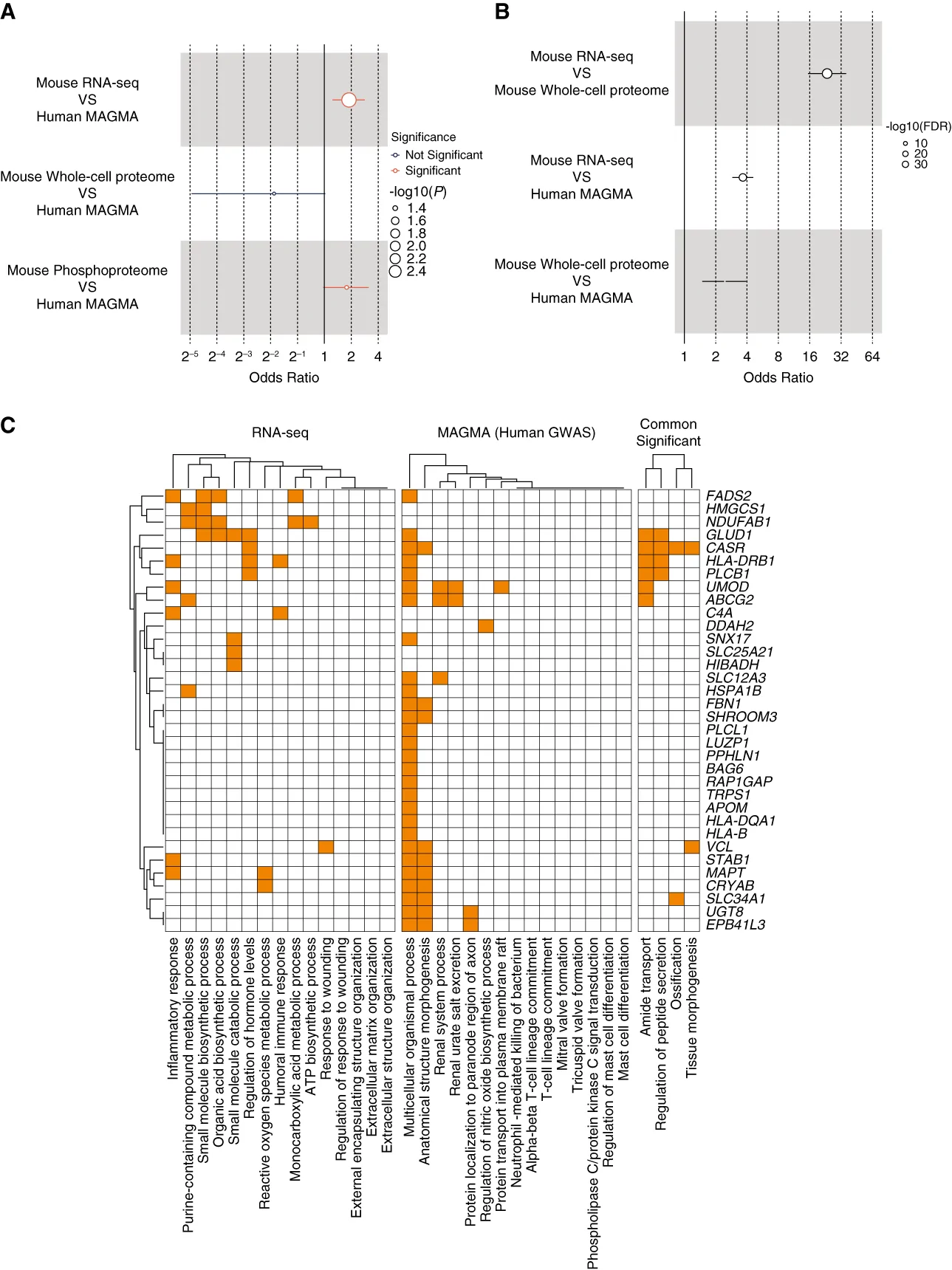
**Integrated analysis between human GWAS data and hyperoxaluric mouse model data.** (A) Overlap between human urolithiasis GWAS gene enrichment results obtained by MAGMA and mouse differential expression analyses across multi-omic modalities. Enrichment was evaluated using the Fisher exact test. The *x* axis represents the OR on a log2 scale. Point size corresponds to the −log10(*P* value). This analysis demonstrates significant enrichment of human results in mouse RNA-seq and phosphoproteome results. Significance indicates passing an FDR threshold of 0.05. (B) Overlap between mouse and human GO biologic process gene set enrichment results. Mouse GO enrichment was derived from gene set enrichment analysis of mouse differential expression results, while human GO enrichment was obtained from MAGMA-based gene set analysis of human GWAS. Enrichment was assessed using Fisher exact test, with the *x* axis showing log2-transformed ORs and point size indicating −log10(FDR). All displayed results met the FDR threshold of 0.05; therefore, statistical significance is not shown. (C) Heatmap illustrates the relationship between cross-species significant genes and enriched GO biological process terms. Columns represent the top 15 GO biological process terms from mouse RNA-seq gene set enrichment analysis, the top 15 GO biological process terms from human MAGMA gene set enrichment analysis, and four GO biological process terms that were significantly enriched in both analyses. Rows correspond to the 46 genes that were significant in both mice and human analyses. Orange cells indicate membership of a gene in the corresponding GO biological process term. GO, gene ontology; OR, odds ratio.

Building on the molecule-level overlap, we next evaluated cross-species concordance at the functional level using gene set enrichment analysis. Comparison of enriched GO biological process terms revealed four terms that were significantly enriched in both mouse and human analyses: regulation of peptide secretion, amide transport, ossification, and tissue morphogenesis (Supplemental Table 3). To quantify the overlap of enriched GO biological process terms between analyses, we applied the Fisher exact test (Figure 3B). This analysis demonstrated highly significant overlap between RNA-seq data from the mouse kidney and human GWAS results (OR=3.7, *P* = 3.2×10^−25^), as well as between the WC proteome and human GWAS results (OR=2.5, *P* = 3.5×10^−4^). In addition, a strong concordance was observed between mouse RNA-seq and WC proteome enrichment results (OR=23.6, *P* = 3.4×10^−36^). Following this, we visualized the assignment of the 46 cross-species urolithiasis-related molecules to enriched GO biological process terms using a heatmap (Figure 3C). Specifically, the heatmap displays GO terms ranked among the top 15 by *P* value in the mouse kidney RNA-seq and human MAGMA analyses, as well as the four GO terms that were significantly enriched in both species. This visualization illustrates where the cross-species–prioritized molecules are positioned within these biologically relevant processes. Overall, 34 of the 46 molecules were assigned to at least one of the displayed GO terms. No GO terms reached statistical significance in the phosphoproteome.

### Cell Type Enrichment Analysis Highlights Important Cell Populations in Urolithiasis

To identify kidney cell types potentially associated with urolithiasis, we performed cell type enrichment analyses using publicly available single-cell RNA-seq data from mouse kidneys^24^ as a reference. Based on this dataset, cells were classified into proximal tubular cells, distal tubular cells, collecting duct intercalated cells (ICs), collecting duct principal cell (PC), stromal cells, immune cells, and others (Figure 4A). We calculated pseudo-bulk expression profiles and tau scores from the single-cell RNA-seq data to define cell type–specific genes, with genes showing tau ≥0.8 considered cell type–specific.^35,36^ These cell type–specific gene sets were then used to assess enrichment in the hyperoxaluric mouse RNA-seq data and the human urolithiasis GWAS results.

**Figure 4:**
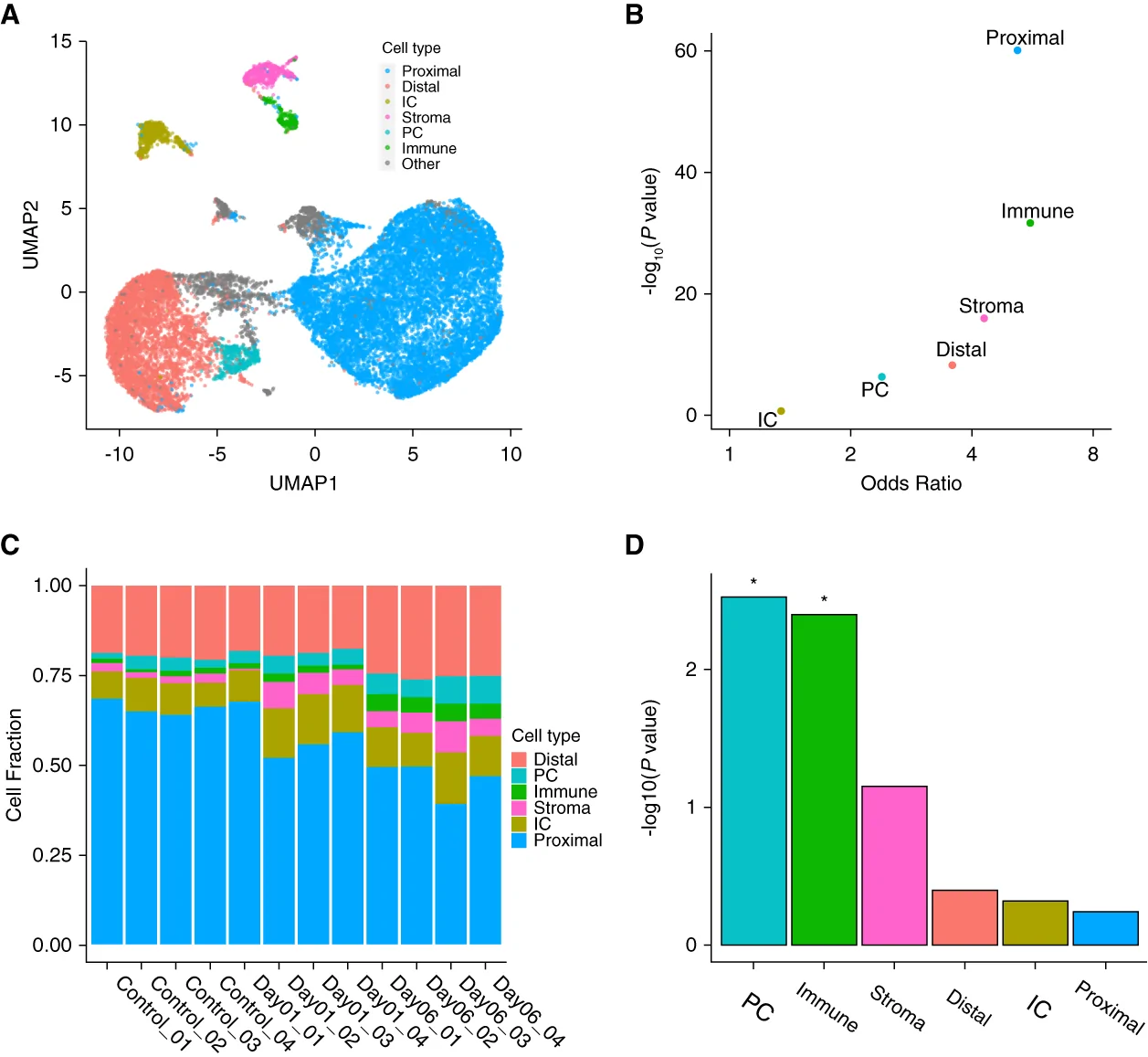
**Cell type-specific analysis of hyperoxaluric mouse model and human urolithiasis GWAS.** (A) UMAP projection of adult mouse kidney single-cell RNA-seq data that are publicly available. The original dataset included a mixture of cells from adult and neonatal mice. For this study, we retained only adult mouse cells for analysis and redefined cell type annotations accordingly. (B) We evaluated whether specific genes in the mouse kidney RNA-seq data from the hyperoxaluric mouse model were enriched in the differentially expressed genes using the Fisher exact test. The *x* axis displays the OR from the Fisher exact test, while the *y* axis shows the negative logarithm of the *P* value. (C) Cell type deconvolution analysis estimated cell fraction for each mouse sample. Cell type deconvolution analysis estimated cell fraction for each mouse sample, revealing an increase in the proportion of Proximal cells, whereas the fractions of other cell types tended to decrease correspondingly. (D) Cell type enrichment analysis using scDRS applied directly to the mouse kidney single-cell RNA-seq data. The bar plot illustrates the degree of enrichment (in terms of −log10 *P* values) of genetic risk for urolithiasis across redefined kidney cell types. *False discovery rate (Benjamini-Hochberg-adjusted *P* value) < 0.05. PC and Immune showed significance after considering multiple test correlation. Distal, distal tubular cells; IC, intercalated cell; Immune, immune cells; PC, principal cell; Proximal, proximal tubular cells; scDRS, single-cell disease relevance score; Stroma, stromal cells; UMAP, uniform manifold approximation and projection.

The Fisher exact test was used to evaluate which cell type–specific genes were more abundant in the differentially expressed genes of the mouse RNA-seq data. Genes expressed in specific cell types other than collecting duct ICs were enriched among the differentially expressed genes in the mouse RNA-seq data (Figure 4B). The *P* values for proximal tubular cells, immune cells, stroma cells, distal tubule cells, and collecting duct PCs were 8.0×10^−61^, 2.0×10^−32^, 9.5×10^−17^, 5.3×10^−9^, and 4.6×10^−7^, respectively. Next, to assess changes in cell composition following glyoxylate administration, we performed cell type deconvolution analysis on the mouse bulk RNA-seq data using CIBERSORTx with the mouse single-cell RNA-seq dataset as a reference. This analysis revealed that, as the duration of glyoxylate administration increased, the proportion of proximal tubular cells decreased, while the proportions of other cell types tended to increase (Figure 4C).

According to calculations using scDRS,^23^ the *P* values for collecting duct PCs, immune system cells, stroma cells, distal tubular cells, collecting duct ICs, and proximal tubular cells were 3.0×10^−3^, 4.0×10^−3^, 0.071, 0.40, 0.48, and 0.58, respectively (Figure 4D). Even after multiple testing corrections, collecting duct PCs and immune system cells remained significantly enriched. As this study aimed to integrate mouse experimental data with human GWAS results using the same single-cell reference, we employed the mouse single-cell dataset described above. To complement these analyses and directly link GWAS findings with human kidney biology, we further incorporated human single-cell datasets. We also attempted cell type enrichment analyses of human GWAS data using the KPMP human kidney single-cell RNA-seq dataset (Supplemental Figure 4, A and B) with linkage disequilibrium score regression specifically expressed gene and scDRS; however, neither approach revealed significant enrichment in any specific cell type (Supplemental Figure 4, C and D). These results indicate that the essential cell types for urolithiasis in both mice and humans are immune cells and collecting duct PCs, which accounted for six of 31 genes identified through cross-species analysis (Table 1).

### Follow-Up Analysis of Cross-Species Urolithiasis-Related Molecules Using Human Renal Papilla Gene Expression and Kidney eQTL Data

As shown in Table 1, we identified 46 molecules potentially related to urolithiasis. To assess their relevance to human disease, we performed follow-up analysis using gene expression data from human renal papillae and colocalization and MR analysis using kidney eQTL data.

The renal papilla is located near the openings of the collecting ducts into the urinary tract, where a kidney stone initially forms.^37^ To assess whether the 46 cross-species urolithiasis-related molecules identified in our integrative analysis are also transcriptionally altered in the renal papilla, we analyzed a microarray-based transcriptome dataset^27^ comparing renal papillae with and without stones from patients with nephrolithiasis. Stones formed at this site are known as Randall's plaques. In the differential expression analysis, eight out of the 46 cross-species urolithiasis-related molecules exhibited *P* < 0.05, and considering multiple testing, *SHROOM3* (log-fold change=0.43, *P* = 4.0×10^−4^) and *CRYAB* (log-fold change=1.3, *P* = 1.0×10^−3^) exhibited FDR values <0.05 (Figure 5A). *CRYAB* and *SHROOM3* showed consistent upregulation in mouse and human gene expression data. We conducted a permutation test using random shuffling of gene labels to demonstrate that these results were not obtained by chance (Supplemental Figure 5; see Supplemental Methods: permutation analysis). The empirical *P* value for observing two genes from collecting duct PCs or proximal tubular cells with FDR <0.05 was 0.0066, all of which were significant.

**Figure 5:**
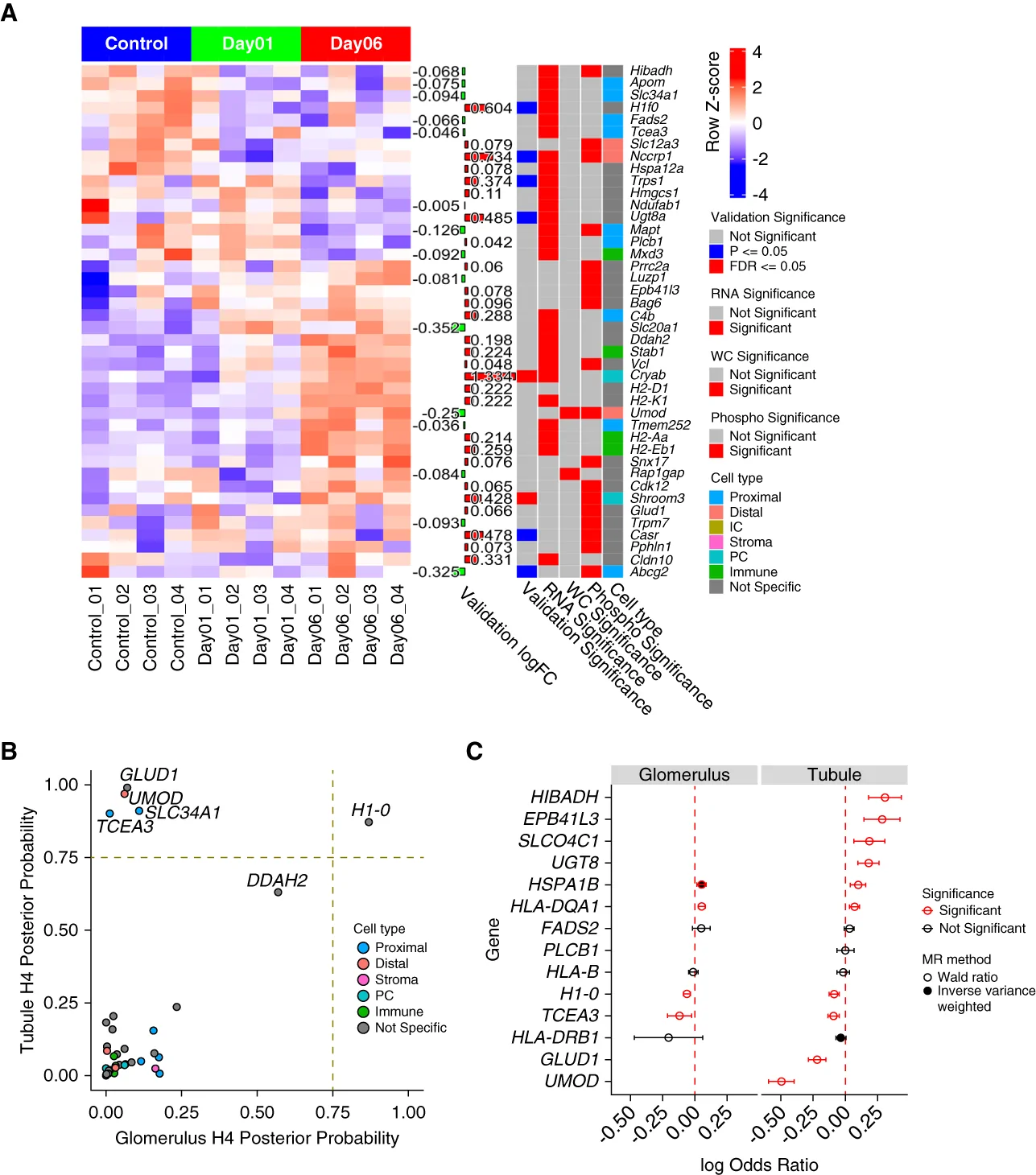
**Follow-up analyses of cross-species genes using human renal expression and GWAS colocalization analyses.** (A) The cross-species urolithiasis-related molecules that were also quantified in the gene expression data of the human renal papillae are shown. Heatmap provides a visual representation of gene expression profiles derived from RNA-seq data in mice. The first and second columns of the row annotation display the findings of this follow-up analysis, which involved examining gene expression in the renal papillae of humans with and without kidney stones. The first column illustrates the log FC, whereas the second column represents the level of significance. In the significance column, blue indicates *P* values lower than 0.05, whereas red signifies FDR values below 0.05. Cell types were from adult mouse kidney single-cell RNA-seq data. (B) The plot depicts the outcomes of the colocalization analysis for urolithiasis GWAS and renal eQTL data across urolithiasis-associated gene regions from cross-species multi-omics analysis. The *x* axis represents the colocalization result between urolithiasis GWAS and glomerulus eQTL, and the *y* axis represents the colocalization result between urolithiasis GWAS and tubule eQTL data. The graph displays the posterior probability of H4, which suggests that the same variant is responsible for causing both urolithiasis GWAS and kidney eQTL. The significance level is set at a posterior probability of 0.75 or higher. The colors of points represent cell types determined from adult mouse kidney single-cell RNA-seq data. (C) MR analysis was performed to assess whether the eQTL data at the chromosomal regions of the cross-species urolithiasis-related molecules suggest a causal effect on urolithiasis. The *x* axis represents the estimated log ORs, and the *y* axis indicates the gene names corresponding to the examined regions. Phenotypes with an FDR of <0.05 were considered significant. Regions lacking variants with a *P* value ≤ 5.0×10^−8^ in the eQTL dataset were excluded from the analysis. eQTL, expression quantitative trait loci; FC, fold-change; H4, hypothesis 4; MR, Mendelian randomization.

We performed colocalization analysis to examine whether urolithiasis GWAS signals and kidney eQTL signals overlapped within the chromosomal regions of the 46 cross-species urolithiasis-related molecules. We used the publicly available kidney eQTL dataset from NephQTL2.^38^ This dataset includes eQTL data from both the tubular and glomerular regions. Notably, *GLUD1*, *UMOD*, *SLC34A1*, *TCEA3*, and *H1-0* demonstrated H4 posterior probabilities from tubular eQTL data exceeding 0.75, with values of 0.99, 0.98, 0.91, 0.90, and 0.87, respectively (Figure 5B). For glomerular eQTL, only *H1-0* showed a significant H4 posterior probability (0.87). In this analysis, it was expected that molecules derived from proximal and distal tubules in the cell type enrichment analysis would colocalize with tubular eQTL data. Because *UMOD* is derived from distal tubules and *SLC34A1* and *TCEA3* are derived from proximal tubules, these molecules can be considered anatomically validated.

Furthermore, we investigated whether kidney eQTLs within the chromosomal regions of the 46 cross-species urolithiasis-related molecules were associated with urolithiasis in the human GWAS summary statistics. Consistent with the colocalization findings, MR analysis indicated that genetically predicted expression of the genes in the tubular region; *UMOD* (log OR: −0.50, *P* = 2.6×10^−22^), *GLUD1* (log OR: −0.22, *P* = 1.6×10^−10^), *TCEA3* (log OR: −0.091, *P* = 5.2×10^−5^), and *H1-0* (log OR: −0.086, *P* = 9.54×10^−6^) as well as *H1-0* (log OR: −0.061, *P* = 9.1×10^−6^) in the glomerular region (Figure 5C).

Together, these analyses demonstrate that several cross-species urolithiasis-related molecules, including *CRYAB*, *SHROOM3*, *UMOD*, *GLUD1*, *SLC34A1*, *TCEA3*, and *H1-0*, are consistently supported by human renal papilla gene expression, kidney eQTL colocalization, and MR analyses.

## Discussion

Although expression-based molecular data from a hyperoxaluric mouse model or genetic association data from GWAS alone are insufficient to reveal the pathophysiology of urolithiasis, we aimed to explore converging evidence from both sources to propose candidate molecules potentially involved in disease mechanisms. Multi-omics analysis of mouse kidneys revealed numerous differentially expressed molecules. Gene set enrichment analysis using GO biological process revealed distinct biologic themes underlying nephrocalcinosis in hyperoxaluric mice. GO biological process enrichment highlighted inflammatory and injury-related responses, along with extensive metabolic reprogramming, including alterations in small-molecule, lipid, and energy metabolism. These findings are consistent with the notion that calcium oxalate deposition induces local tissue injury and inflammation, accompanied by adaptive metabolic changes in renal tubular cells.^13,39^

Gene enrichment analysis of human urolithiasis GWAS identified 343 significant genes, providing a gene-level framework for disease susceptibility. Subsequent MAGMA-based gene set enrichment analysis further revealed that these genes are clustered within specific GO Biological Process categories. Interestingly, the appearance of immune-related pathways such as T-cell lineage commitment and neutrophil-mediated responses in the gene set enrichment results of a GWAS—a study design that is inherently independent of acute acquired factors—highlights a potential genetic predisposition of immune regulation in urolithiasis susceptibility. Moreover, this finding is consistent with previous reports linking urolithiasis to immune-related mechanisms.^40^

Cross-species analyses have been frequently employed in previous studies and have sometimes yielded meaningful results.^41^ In this study, we identified 46 cross-species urolithiasis-related molecules that showed differential expression in a hyperoxaluric mouse model and were supported by GWAS data. Gene set enrichment analysis revealed four GO biological process terms that were significantly enriched in both human GWAS and the mouse model: regulation of peptide secretion, amide transport, ossification, and tissue morphogenesis. Notably, the shared enrichment of ossification—a process directly related to calcium mineralization—is particularly intriguing in the context of urolithiasis, a disease characterized by pathologic crystal formation. These shared gene set terms also provide functional context for interpreting the cross-species urolithiasis-related molecules. For example, *CASR* and *SLC34A1*, both assigned to the ossification category, are well known to play key roles in calcium homeostasis: pathogenic variants in *CASR* cause Bartter syndrome^42–44^ with nephrocalcinosis, whereas dysfunction of *SLC34A1* has been implicated in infantile hypercalcemia.^45^ Together, these observations suggest that cross-species–enriched biologic processes help us to interpret cross-species urolithiasis-related molecules.

Cell type enrichment analyses using single-cell RNA-seq data of the mouse kidney suggested that immune cells and collecting duct PCs may be involved in urolithiasis, based on enrichment signals observed in both mouse and human datasets. Several studies have suggested the involvement of immune cells, including macrophages, in urolithiasis.^46–48^ However, when we performed cell type enrichment analyses using human kidney single-cell RNA-seq data as a reference, no significant enrichment was observed.

Possible explanations for this discrepancy include differences in cell type composition between the reference datasets. The mouse data were derived from the whole kidney, whereas the human samples were obtained by cold-cup biopsy. Proximal tubule-derived transcripts typically dominate bulk kidney RNA^49^; however, the biopsy samples may deviate from this composition because of the anatomic specification. Therefore, to enable consistent comparisons across species, we primarily relied on mouse single-cell data for cell type enrichment analyses.

In a follow-up analysis using gene expression data from renal papillae samples of urolithiasis patients, *SHROOM3* and *CRYAB* were found to be significantly upregulated in regions with Randall plaque compared with adjacent stone-free regions. Because Randall plaques typically form near the openings of the collecting ducts on the papillary surface, cells derived from the collecting ducts are anatomically positioned to influence plaque formation. Because collecting duct PCs were highlighted in both human and mouse data by the cell type enrichment analysis, molecules derived from these cells are likely to be differentially expressed around Randall plaques. *SHROOM3* and *CRYAB* are predominantly expressed in collecting duct PCs. *SHROOM3* is a gene implicated in CKD and is known to be involved in cellular morphology.^50^ It is conceivable that alterations in cell adhesion mediated by *SHROOM3* could affect molecular reabsorption, potentially linking it to urolithiasis.

Both colocalization and MR analyses consistently identified *UMOD*, *GLUD1*, *TCEA3*, and *H1-0* as significantly associated with urolithiasis, supporting their strong involvement in disease pathogenesis. *UMOD* encodes a glycoprotein secreted from the thick ascending limb of Henle loop into the tubular lumen and has been implicated not only in urolithiasis but also in renal function and vascular calcification suppression.^51^ In our analysis, *UMOD* was classified as distal tubule–specific, consistent with our categorization of the thick ascending limb as a distal nephron compartment (see Supplemental Methods: processing of single-cell RNA-seq reference datasets). The role of *GLUD1* in the kidney with respect to urolithiasis remains largely unclear. However, *GLUD1* was assigned to two of the four GO biological process terms that were commonly enriched across human and mouse results—regulation of peptide secretion and amide transport—suggesting indirect involvement in kidney stone formation. Notably, *GLUD1* encodes glutamate dehydrogenase, a central enzyme in glutamate metabolism linking amino acid turnover to cellular energy and nitrogen balance. In renal clear cell carcinoma, *GLUD1* downregulation has been shown to activate the phosphoinositide 3-kinase/Akt/mTOR signaling pathway, suggesting that altered *GLUD1* activity in renal cells may modulate intracellular signaling and metabolic adaptation under conditions of oxalate-induced stress.^52^ This interpretation is further supported by our phosphoproteome-based kinase set enrichment analysis, which highlighted mTOR. Furthermore, previous studies have reported that higher urinary L-glutamate levels are associated with lower calcium oxalate crystal concentrations, suggesting a potential protective role for glutamate in the urinary microenvironment.^53^ Taken together, these observations suggest that *GLUD1* may indirectly influence susceptibility to urolithiasis by modulating amino acid metabolism and peptide handling.

This study has limitations. First, the hyperoxaluric mouse model has not been shown to accurately reflect the pathogenesis of human urolithiasis. This model may more closely represent pathologic conditions associated with severe hyperoxaluria, such as primary hyperoxaluria, rather than idiopathic calcium oxalate stone disease. Differentially expressed molecules identified by the mouse kidney multi-omics analysis may partly reflect changes in cell composition in response to glyoxylate, as indicated by cell type deconvolution. Second, the use of a European linkage disequilibrium reference panel in cross-population meta-GWAS analyses may have affected the interpretation of association signals in Japanese cohorts. Third, cross-species comparisons are typically restricted to molecules that have orthologs, potentially excluding species-specific or rapidly evolving loci. Fourth, the gene ID conversion from mouse to human for multi-omics integration may have introduced mapping inaccuracies or excluded nonconserved genes. Details regarding the ortholog mapping procedure, as well as the types of genes that could not be aligned between humans and mice, are provided in Supplemental Methods: Integration of human and mouse data and in Supplemental Data 1. Fifth, cell type enrichment analysis was based on single-cell RNA-seq data from healthy mouse kidneys, which may not reflect the cellular state in the hyperoxaluric model or align directly with human kidney cell types. Finally, differential expression and colocalization analyses cannot establish causal relationships. The results of MR analyses should be interpreted cautiously and do not constitute definitive proof of causality.

In conclusion, this cross-species analysis integrating human urolithiasis GWAS and multi-omics profiling of a hyperoxaluric mouse model prioritized 46 cross-species urolithiasis-related molecules with potential relevance to urolithiasis. These molecules, supported by genetic association signals and expression-based evidence, may contribute to a better understanding of disease mechanisms and serve as a resource for future biomarker or therapeutic target discovery.

## Supplementary Material

## Data Availability

Original data generated for the study are or will be made available in a public access repository upon publication. Data Type: Aggregated Data and Raw Data/Source Data. Repository Name: GEO and Mass Spectrometry Interactive Virtual Environment. Linkable Citation: The complete RNA-Seq dataset is available from the GEO repository (GSE282711). https://www.ncbi.nlm.nih.gov/geo/query/acc.cgi?acc=GSE282711. https://drive.google.com/file/d/1kYWgVrH_ihs0gMw2qkdpIPvclKd1Yxi0/view?usp=sharing. The proteomics datasets used in this study have been deposited in the Mass Spectrometry Interactive Virtual Environment repository (MSV000099680). https://massive.ucsd.edu/ProteoSAFe/private-dataset.jsp?task=b8ea55e733844a5096b91119f74446c9. WC proteomics data: https://drive.google.com/file/d/1QRvekTMFpex0tyFfjGl8uNZ-3E3G7CNz/view?usp=sharing. Phosphoproteomics data: https://drive.google.com/file/d/1l87Ph3kzUokh7bb_3axf_P8GK84Vd5Kd/view?usp=sharing. The GWAS summary statistics used for the analysis were downloaded from the BBJ pheweb (https://pheweb.jp/). Mouse kidney single-cell RNA-seq data were obtained from the GEO repository (GSE157079) and used in this study. The reference single-cell RNA-seq data of human kidneys were obtained from the KPMP (https://www.kpmp.org/). Gene expression data from the renal papillae, obtained using a microarray, were retrieved from the GEO repository (GSE73680). Human kidney eQTL data were obtained from the NephQTL (https://nephqtl.org/). These datasets and their sources are summarized in Supplemental Table 4 for clarity.

## Declarative Statements

All animal experiments were conducted in accordance with the NIH Guide for the Care and Use of Laboratory Animals or an equivalent standard that meets or exceeds the ethical and welfare requirements outlined in the NIH Guide. All protocols were approved by the appropriate institutional animal care and use committee.

## Supplemental Material

Supplemental Methods and References

Supplemental Figure 1. Multi-omics analysis of the kidney from the hyperoxaluric mouse model using the three modalities.

Supplemental Figure 2. Gene set enrichment analysis of the hyperoxaluric model mice.

Supplemental Figure 3. Analysis of urolithiasis GWAS summary statistics.

Supplemental Figure 4. Cell type annotation and enrichment analysis of human kidney scRNA-seq data in urolithiasis GWAS.

Supplemental Figure 5. Follow-up analysis of human renal papilla gene expression data using permutation tests.

Supplemental Table 1. Experimental data for the hyperoxaluric mouse model.

Supplemental Table 2. Human–mouse orthologs of cross-species candidate molecules.

Supplemental Table 3. Shared significantly enriched GO biological process terms between mouse kidney RNA-seq and human GWAS (MAGMA) analyses.

Supplemental Table 4. Summary of publicly available datasets used in this study.
