# Supplementary material for: Cross-Species Comparison between Mouse Kidney Multi-Omics and Human Genome-Wide Association Studies Highlights Molecular Features Associated with Oxalate Nephropathy
Source: Kidney360. 2026 Jun 11;7(9):1968–84. doi: 10.34067/KID.0000001211 (PMC13641535; doi:10.34067/KID.0000001211)
Supplement: Supplementary file 1 [file kidney360-7-1968-s001.pdf]

ASN American Society of Nephrology
JASN Journal of the American Society of Nephrology
KIDNEY360 Accessing Our World From Every Angle
CJASN Clinical Journal of the American Society of Nephrology

ASN Journal Disclosure Form

As per ASN journal policy, I have disclosed any financial relationships or commitments I have held in the past 36 months as included below. I have listed my Current Employer below to indicate there is a relationship requiring disclosure. If no relationship exists, my Current Employer is not listed.

R. Chaya has nothing to disclose.

I understand that the information above will be published within the journal article, if accepted, and that failure to comply and/or to accurately and completely report the potential financial conflicts of interest could lead to the following: 1) Prior to publication, article rejection, or 2) Post-publication, sanctions ranging from, but not limited to, issuing a correction, reporting the inaccurate information to the authors' institution, banning authors from submitting work to ASN journals for varying lengths of time, and/or retraction of the published work.

Name: Ryosuke Chaya
Manuscript ID: K360-2025-000716R1
Manuscript Title: Cross-species comparison between mouse kidney multi-omics and human genome-wide association studies highlights molecular features linked to urolithiasis risk.
Date of Completion: October 20, 2025
Disclosure Updated Date: October 20, 2025

ASN American Society of Nephrology | JASN Journal of the American Society of Nephrology | KIDNEY360 Accessing Our World From Every Angle | CJASN Clinical Journal of the American Society of Nephrology

ASN Journal Disclosure Form

As per ASN journal policy, I have disclosed any financial relationships or commitments I have held in the past 36 months as included below. I have listed my Current Employer below to indicate there is a relationship requiring disclosure. If no relationship exists, my Current Employer is not listed.

I. Cristea reports the following:
Ownership Interest: SAB Member for Evrys Bio, company focused on antiviral drugs; and Patents or Royalties: Patent on "Method and System for Thermal Stabilization of Pharmaceutical Formulations Containing Enveloped and Nonenveloped Virus Particles" - PCT/US25/24093; Patent on "Sirt4 Lipoamidase Activity and Uses Thereof" - US 11,726,088; Patent on "METHOD FOR DETECTION AND QUANTITATIVE MONITORING OF INFECTIONS WITH HERPESVIRUSES"; Patent on "Use of Sirtuin Modulators to Inhibit Virus Growth".

I understand that the information above will be published within the journal article, if accepted, and that failure to comply and/or to accurately and completely report the potential financial conflicts of interest could lead to the following: 1) Prior to publication, article rejection, or 2) Post-publication, sanctions ranging from, but not limited to, issuing a correction, reporting the inaccurate information to the authors' institution, banning authors from submitting work to ASN journals for varying lengths of time, and/or retraction of the published work.

Name: Ileana M. Cristea
Manuscript ID: K360-2025-000716R1
Manuscript Title: Cross-species comparison between mouse kidney multi-omics and human genome-wide association studies highlights molecular features linked to urolithiasis risk
Date of Completion: October 20, 2025
Disclosure Updated Date: October 20, 2025

ASN
American Society of Nephrology
JASN
Journal of the American Society of Nephrology
KIDNEY360
Accessing Our World From Every Angle
CJASN
Clinical Journal of the American Society of Nephrology

ASN Journal Disclosure Form

As per ASN journal policy, I have disclosed any financial relationships or commitments I have held in the past 36 months as included below. I have listed my Current Employer below to indicate there is a relationship requiring disclosure. If no relationship exists, my Current Employer is not listed.

R. Edahiro reports the following:
Employer: The University of Osaka

I understand that the information above will be published within the journal article, if accepted, and that failure to comply and/or to accurately and completely report the potential financial conflicts of interest could lead to the following: 1) Prior to publication, article rejection, or 2) Post-publication, sanctions ranging from, but not limited to, issuing a correction, reporting the inaccurate information to the authors' institution, banning authors from submitting work to ASN journals for varying lengths of time, and/or retraction of the published work.

Name: Ryuya Edahiro
Manuscript ID: K360-2025-000716R4
Manuscript Title: Cross-species comparison between mouse kidney multi-omics and human genome-wide association studies highlights molecular features associated with oxalate nephropathy.
Date of Completion: April 4, 2026
Disclosure Updated Date: February 18, 2026

ASN American Society of Nephrology JASN Journal of the American Society of Nephrology KIDNEY360 Accessing Our World From Every Angle CJASN Clinical Journal of the American Society of Nephrology

ASN Journal Disclosure Form

As per ASN journal policy, I have disclosed any financial relationships or commitments I have held in the past 36 months as included below. I have listed my Current Employer below to indicate there is a relationship requiring disclosure. If no relationship exists, my Current Employer is not listed.

S. Hamamoto reports the following:
Employer: Nagoya City University Graduate School of Medical Sciences; Nagoya City University West Medical Center; Research Funding: Takeda Science Foundation; Casio Science Promotion Foundation;; Honoraria: Olympus Corporation; Johnson & Johnson; Ono Pharmaceutical co. LTD., Becton Dickinson, Boston Scientific,; and Speakers Bureau: Johnson & Johnson; Ono Pharmaceutical co, Olympus Corporation.

I understand that the information above will be published within the journal article, if accepted, and that failure to comply and/or to accurately and completely report the potential financial conflicts of interest could lead to the following: 1) Prior to publication, article rejection, or 2) Post-publication, sanctions ranging from, but not limited to, issuing a correction, reporting the inaccurate information to the authors' institution, banning authors from submitting work to ASN journals for varying lengths of time, and/or retraction of the published work.

Name: Shuzo Hamamoto
Manuscript ID: K360-2025-000716R2
Manuscript Title: Cross-species comparison between mouse kidney multi-omics and human genome-wide association studies highlights molecular features associated with nephrocalcinosis.
Date of Completion: February 17, 2026
Disclosure Updated Date: February 17, 2026

ASN
American Society of Nephrology
JASN
Journal of the American Society of Nephrology
KIDNEY360
Accessing Our World From Every Angle
CJASN
Clinical Journal of the American Society of Nephrology

ASN Journal Disclosure Form

As per ASN journal policy, I have disclosed any financial relationships or commitments I have held in the past 36 months as included below. I have listed my Current Employer below to indicate there is a relationship requiring disclosure. If no relationship exists, my Current Employer is not listed.

Y. Hashimoto reports the following:
Employer: Nagoya City University

I understand that the information above will be published within the journal article, if accepted, and that failure to comply and/or to accurately and completely report the potential financial conflicts of interest could lead to the following: 1) Prior to publication, article rejection, or 2) Post-publication, sanctions ranging from, but not limited to, issuing a correction, reporting the inaccurate information to the authors' institution, banning authors from submitting work to ASN journals for varying lengths of time, and/or retraction of the published work.

Name: Yutaka Hashimoto
Manuscript ID: K360-2025-000716R1
Manuscript Title: Cross-species comparison between mouse kidney multi-omics and human genome-wide association studies highlights molecular features linked to urolithiasis risk.
Date of Completion: October 20, 2025
Disclosure Updated Date: October 20, 2025

ASN
American Society of Nephrology
JASN
Journal of the American Society of Nephrology
KIDNEY360
Accessing Our World From Every Angle
CJASN
Clinical Journal of the American Society of Nephrology

ASN Journal Disclosure Form

As per ASN journal policy, I have disclosed any financial relationships or commitments I have held in the past 36 months as included below. I have listed my Current Employer below to indicate there is a relationship requiring disclosure. If no relationship exists, my Current Employer is not listed.

Y. Kato has nothing to disclose.

I understand that the information above will be published within the journal article, if accepted, and that failure to comply and/or to accurately and completely report the potential financial conflicts of interest could lead to the following: 1) Prior to publication, article rejection, or 2) Post-publication, sanctions ranging from, but not limited to, issuing a correction, reporting the inaccurate information to the authors' institution, banning authors from submitting work to ASN journals for varying lengths of time, and/or retraction of the published work.

Name: Yoichi Kato
Manuscript ID: K360-2025-000716R1
Manuscript Title: Cross-species comparison between mouse kidney multi-omics and human genome-wide association studies highlights molecular features linked to urolithiasis risk
Date of Completion: October 20, 2025
Disclosure Updated Date: October 20, 2025

ASN American Society of Nephrology JASN Journal of the American Society of Nephrology KIDNEY360 Accessing Our World From Every Angle CJASN Clinical Journal of the American Society of Nephrology

ASN Journal Disclosure Form

As per ASN journal policy, I have disclosed any financial relationships or commitments I have held in the past 36 months as included below. I have listed my Current Employer below to indicate there is a relationship requiring disclosure. If no relationship exists, my Current Employer is not listed.

J. Muroski has nothing to disclose.

I understand that the information above will be published within the journal article, if accepted, and that failure to comply and/or to accurately and completely report the potential financial conflicts of interest could lead to the following: 1) Prior to publication, article rejection, or 2) Post-publication, sanctions ranging from, but not limited to, issuing a correction, reporting the inaccurate information to the authors' institution, banning authors from submitting work to ASN journals for varying lengths of time, and/or retraction of the published work.

Name: John M Muroski
Manuscript ID: K360-2025-000716R1
Manuscript Title: Cross-species comparison between mouse kidney multi-omics and human genome-wide association studies highlights molecular features linked to urolithiasis risk.
Date of Completion: October 31, 2025
Disclosure Updated Date: October 31, 2025

ASN American Society of Nephrology | JASN Journal of the American Society of Nephrology | KIDNEY360 Accessing Our World From Every Angle | CJASN Clinical Journal of the American Society of Nephrology

ASN Journal Disclosure Form

As per ASN journal policy, I have disclosed any financial relationships or commitments I have held in the past 36 months as included below. I have listed my Current Employer below to indicate there is a relationship requiring disclosure. If no relationship exists, my Current Employer is not listed.

S. Namba has nothing to disclose.

I understand that the information above will be published within the journal article, if accepted, and that failure to comply and/or to accurately and completely report the potential financial conflicts of interest could lead to the following: 1) Prior to publication, article rejection, or 2) Post-publication, sanctions ranging from, but not limited to, issuing a correction, reporting the inaccurate information to the authors' institution, banning authors from submitting work to ASN journals for varying lengths of time, and/or retraction of the published work.

Name: Shinichi Namba
Manuscript ID: K360-2025-000716R1
Manuscript Title: Cross-species comparison between mouse kidney multi-omics and human genome-wide association studies highlights molecular features linked to urolithiasis risk.
Date of Completion: October 20, 2025
Disclosure Updated Date: October 20, 2025

ASN Journal Disclosure Form

As per ASN journal policy, I have disclosed any financial relationships or commitments I have held in the past 36 months as included below. I have listed my Current Employer below to indicate there is a relationship requiring disclosure. If no relationship exists, my Current Employer is not listed.

A. Okada reports the following:
Employer: Nagoya City University Graduate School of Medical Sciences

I understand that the information above will be published within the journal article, if accepted, and that failure to comply and/or to accurately and completely report the potential financial conflicts of interest could lead to the following: 1) Prior to publication, article rejection, or 2) Post-publication, sanctions ranging from, but not limited to, issuing a correction, reporting the inaccurate information to the authors' institution, banning authors from submitting work to ASN journals for varying lengths of time, and/or retraction of the published work.

Name: Atsushi Okada
Manuscript ID: K360-2025-000716R3
Manuscript Title: Cross-species comparison between mouse kidney multi-omics and human genome-wide association studies highlights molecular features associated with oxalate nephropathy.
Date of Completion: March 24, 2026
Disclosure Updated Date: October 20, 2025

ASN
American Society of Nephrology
JASN
Journal of the American Society of Nephrology
KIDNEY360
Accessing Our World From Every Angle
CJASN
Clinical Journal of the American Society of Nephrology

ASN Journal Disclosure Form

As per ASN journal policy, I have disclosed any financial relationships or commitments I have held in the past 36 months as included below. I have listed my Current Employer below to indicate there is a relationship requiring disclosure. If no relationship exists, my Current Employer is not listed.

Y. Okada has nothing to disclose.

I understand that the information above will be published within the journal article, if accepted, and that failure to comply and/or to accurately and completely report the potential financial conflicts of interest could lead to the following: 1) Prior to publication, article rejection, or 2) Post-publication, sanctions ranging from, but not limited to, issuing a correction, reporting the inaccurate information to the authors' institution, banning authors from submitting work to ASN journals for varying lengths of time, and/or retraction of the published work.

Name: Yukinori Okada
Manuscript ID: K360-2025-000716R5
Manuscript Title: Cross-species comparison between mouse kidney multi-omics and human genome-wide association studies highlights molecular features associated with oxalate nephropathy.
Date of Completion: May 23, 2026
Disclosure Updated Date: May 23, 2026

ASN American Society of Nephrology
JASN Journal of the American Society of Nephrology
KIDNEY360 Accessing Our World From Every Angle
CJASN Clinical Journal of the American Society of Nephrology

ASN Journal Disclosure Form

As per ASN journal policy, I have disclosed any financial relationships or commitments I have held in the past 36 months as included below. I have listed my Current Employer below to indicate there is a relationship requiring disclosure. If no relationship exists, my Current Employer is not listed.

N. Sasa has nothing to disclose.

I understand that the information above will be published within the journal article, if accepted, and that failure to comply and/or to accurately and completely report the potential financial conflicts of interest could lead to the following: 1) Prior to publication, article rejection, or 2) Post-publication, sanctions ranging from, but not limited to, issuing a correction, reporting the inaccurate information to the authors' institution, banning authors from submitting work to ASN journals for varying lengths of time, and/or retraction of the published work.

Name: Noah Sasa
Manuscript ID: K360-2025-000716R1
Manuscript Title: "Cross-species comparison between mouse kidney multi-omics and human genome-wide association studies highlights molecular features linked to urolithiasis risk."
Date of Completion: October 22, 2025
Disclosure Updated Date: October 22, 2025

ASN Journal Disclosure Form

As per ASN journal policy, I have disclosed any financial relationships or commitments I have held in the past 36 months as included below. I have listed my Current Employer below to indicate there is a relationship requiring disclosure. If no relationship exists, my Current Employer is not listed.

Y. Shirai reports the following:
Employer: The University of Osaka

I understand that the information above will be published within the journal article, if accepted, and that failure to comply and/or to accurately and completely report the potential financial conflicts of interest could lead to the following: 1) Prior to publication, article rejection, or 2) Post-publication, sanctions ranging from, but not limited to, issuing a correction, reporting the inaccurate information to the authors' institution, banning authors from submitting work to ASN journals for varying lengths of time, and/or retraction of the published work.

Name: Yuya Shirai
Manuscript ID: K360-2025-000716R1
Manuscript Title: Cross-species comparison between mouse kidney multi-omics and human genome-wide association studies highlights molecular features linked to urolithiasis risk.
Date of Completion: October 20, 2025
Disclosure Updated Date: October 20, 2025

ASN Journal Disclosure Form

As per ASN journal policy, I have disclosed any financial relationships or commitments I have held in the past 36 months as included below. I have listed my Current Employer below to indicate there is a relationship requiring disclosure. If no relationship exists, my Current Employer is not listed.

K. Sonehara has nothing to disclose.

I understand that the information above will be published within the journal article, if accepted, and that failure to comply and/or to accurately and completely report the potential financial conflicts of interest could lead to the following: 1) Prior to publication, article rejection, or 2) Post-publication, sanctions ranging from, but not limited to, issuing a correction, reporting the inaccurate information to the authors' institution, banning authors from submitting work to ASN journals for varying lengths of time, and/or retraction of the published work.

Name: Kyuto Sonehara
Manuscript ID: K360-2025-000716R1
Manuscript Title: Cross-species comparison between mouse kidney multi-omics and human genome-wide association studies highlights molecular features linked to urolithiasis risk.
Date of Completion: October 20, 2025
Disclosure Updated Date: October 20, 2025

ASN American Society of Nephrology JASN Journal of the American Society of Nephrology KIDNEY360 Accessing Our World From Every Angle CJASN Clinical Journal of the American Society of Nephrology

ASN Journal Disclosure Form

As per ASN journal policy, I have disclosed any financial relationships or commitments I have held in the past 36 months as included below. I have listed my Current Employer below to indicate there is a relationship requiring disclosure. If no relationship exists, my Current Employer is not listed.

K. Taguchi reports the following:
Consultancy: Becton Dickinson; Dornier MedTech America; Verve Meical;

I understand that the information above will be published within the journal article, if accepted, and that failure to comply and/or to accurately and completely report the potential financial conflicts of interest could lead to the following: 1) Prior to publication, article rejection, or 2) Post-publication, sanctions ranging from, but not limited to, issuing a correction, reporting the inaccurate information to the authors' institution, banning authors from submitting work to ASN journals for varying lengths of time, and/or retraction of the published work.

Name: Kazumi Taguchi
Manuscript ID: K360-2025-000716R1
Manuscript Title: Cross-species comparison between mouse kidney multi-omics and human genome-wide association studies highlights molecular features linked to urolithiasis risk.
Date of Completion: October 20, 2025
Disclosure Updated Date: October 20, 2025

ASN American Society of Nephrology
JASN Journal of the American Society of Nephrology
KIDNEY360 Accessing Our World From Every Angle
CJASN Clinical Journal of the American Society of Nephrology

ASN Journal Disclosure Form

As per ASN journal policy, I have disclosed any financial relationships or commitments I have held in the past 36 months as included below. I have listed my Current Employer below to indicate there is a relationship requiring disclosure. If no relationship exists, my Current Employer is not listed.

Y. Tomofuji has nothing to disclose.

I understand that the information above will be published within the journal article, if accepted, and that failure to comply and/or to accurately and completely report the potential financial conflicts of interest could lead to the following: 1) Prior to publication, article rejection, or 2) Post-publication, sanctions ranging from, but not limited to, issuing a correction, reporting the inaccurate information to the authors' institution, banning authors from submitting work to ASN journals for varying lengths of time, and/or retraction of the published work.

Name: Yoshihiko Tomofuji
Manuscript ID: K360-2025-000716R1
Manuscript Title: Cross-species comparison between mouse kidney multi-omics and human genome-wide association studies highlights molecular features linked to urolithiasis risk.
Date of Completion: October 21, 2025
Disclosure Updated Date: October 21, 2025

ASN American Society of Nephrology
JASN Journal of the American Society of Nephrology
KIDNEY360 Accessing Our World From Every Angle
CJASN Clinical Journal of the American Society of Nephrology

ASN Journal Disclosure Form

As per ASN journal policy, I have disclosed any financial relationships or commitments I have held in the past 36 months as included below. I have listed my Current Employer below to indicate there is a relationship requiring disclosure. If no relationship exists, my Current Employer is not listed.

R. Unno reports the following:
Employer: Nagoya City University

I understand that the information above will be published within the journal article, if accepted, and that failure to comply and/or to accurately and completely report the potential financial conflicts of interest could lead to the following: 1) Prior to publication, article rejection, or 2) Post-publication, sanctions ranging from, but not limited to, issuing a correction, reporting the inaccurate information to the authors' institution, banning authors from submitting work to ASN journals for varying lengths of time, and/or retraction of the published work.

Name: Rei Unno
Manuscript ID: K360-2025-000716R5
Manuscript Title: Cross-species comparison between mouse kidney multi-omics and human genome-wide association studies highlights molecular features associated with oxalate nephropathy
Date of Completion: May 18, 2026
Disclosure Updated Date: February 20, 2026

ASN
American Society of Nephrology
JASN
Journal of the American Society of Nephrology
KIDNEY360
Accessing Our World From Every Angle
CJASN
Clinical Journal of the American Society of Nephrology

ASN Journal Disclosure Form

As per ASN journal policy, I have disclosed any financial relationships or commitments I have held in the past 36 months as included below. I have listed my Current Employer below to indicate there is a relationship requiring disclosure. If no relationship exists, my Current Employer is not listed.

K. Yamamoto has nothing to disclose.

I understand that the information above will be published within the journal article, if accepted, and that failure to comply and/or to accurately and completely report the potential financial conflicts of interest could lead to the following: 1) Prior to publication, article rejection, or 2) Post-publication, sanctions ranging from, but not limited to, issuing a correction, reporting the inaccurate information to the authors' institution, banning authors from submitting work to ASN journals for varying lengths of time, and/or retraction of the published work.

Name: Kenichi Yamamoto
Manuscript ID: K360-2025-000716R1
Manuscript Title: Cross-species comparison between mouse kidney multi-omics and human genome-wide association studies highlights molecular features linked to urolithiasis risk.
Date of Completion: October 22, 2025
Disclosure Updated Date: October 22, 2025

ASN Journal Disclosure Form

As per ASN journal policy, I have disclosed any financial relationships or commitments I have held in the past 36 months as included below. I have listed my Current Employer below to indicate there is a relationship requiring disclosure. If no relationship exists, my Current Employer is not listed.

T. Yasui reports the following:
Employer: Nagoya City University Graduate School; Research Funding: Otsuka Pharmaceutical Co., Ltd., Taiho Pharmaceutical Co., Ltd., Chugai Pharmaceutical Co., Ltd., Kyowa Kirin Co., Ltd., Teijin Pharma Limited, Nippon Kayaku Co., Ltd., Nippon Shinyaku Co., Ltd., Boston Scientific Japan K.K, UHA Mikakuto Co., Ltd.;; Honoraria: Igaku-Shoin Ltd., AstraZeneca K.K., Takeda Pharmaceutical Company Limited, Astellas Pharma Inc., Kissei Pharmaceutical Co., Ltd., Janssen Pharmaceutical K.K., Bayer Yakuhin, Ltd., Nippon Kayaku Co., Ltd., Olympus Marketing, Inc., Eisai Co., Ltd., Intuitive Surgical G.K., Ambu K.K., Fuji Yakuhin Co., Ltd., Coloplast K.K., Johnson & Johnson, ONO PHARMACEUTICAL CO., LTD., Boston Scientific Japan K.K.; and Speakers Bureau: AstraZeneca K.K., Takeda Pharmaceutical Company Limited, Astellas Pharma Inc., Kissei Pharmaceutical Co., Ltd., Janssen Pharmaceutical K.K., Bayer Yakuhin, Ltd., Nippon Kayaku Co., Ltd., Olympus Marketing, Inc., Eisai Co., Ltd., Intuitive Surgical G.K., Ambu K.K., Fuji Yakuhin Co., Ltd., Coloplast K.K., Johnson & Johnson, ONO PHARMACEUTICAL CO., LTD., Boston Scientific Japan K.K.

I understand that the information above will be published within the journal article, if accepted, and that failure to comply and/or to accurately and completely report the potential financial conflicts of interest could lead to the following: 1) Prior to publication, article rejection, or 2) Post-publication, sanctions ranging from, but not limited to, issuing a correction, reporting the inaccurate information to the authors' institution, banning authors from submitting work to ASN journals for varying lengths of time, and/or retraction of the published work.

Name: Takahiro Yasui
Manuscript ID: K360-2025-000716R1
Manuscript Title: Cross-species comparison between mouse kidney multi-omics and human genome-wide association studies highlights molecular features linked to urolithiasis risk
Date of Completion: October 20, 2025
Disclosure Updated Date: October 20, 2025
