# Supplementary material for: Cross-Species Comparison between Mouse Kidney Multi-Omics and Human Genome-Wide Association Studies Highlights Molecular Features Associated with Oxalate Nephropathy
Source: Kidney360. 2026 Jun 11;7(9):1968–84. doi: 10.34067/KID.0000001211 (PMC13641535; doi:10.34067/KID.0000001211)
Supplement: Supplementary file 2 [file kidney360-7-1968-s002.pdf]

Supplement to:

Cross-species comparison between mouse kidney multi-omics and human genome-wide association studies highlights molecular features associated with oxalate nephropathy.

Ryosuke Chaya[1,2], Kazumi Taguchi[3*], Yutaka Hashimoto[4], Yuya shirai[2,5], Ryuya Edahiro[2,5,6], Noah Sasa[2,6,7,8], Kenichi Yamamoto[2,9], Yoshihiro Tomofuji[2,6,8], Shinichi Namba[2,6,8], Kyuto Sonehara[2,6,8], Rei Unno[1], Shuzo Hamamoto[1], Atsushi Okada[1], Yoichi Kato[4], John Muroski[10], Ileana M Cristea[10], Yukinori Okada[2,6,8,11,12*], Takahiro Yasui[1]

1. Department of Nephro-urology, Nagoya City University Graduate School of Medical Sciences, Nagoya, Aichi 467-8601 Japan.
2. Department of Statistical Genetics, Osaka University Graduate School of Medicine, Suita 565-0871, Japan.
3. Department of Urology, University of Alabama at Birmingham, Birmingham, Alabama, USA.
4. Department of Cell Biology, Nagoya City University Graduate School of Medical Sciences, Nagoya, Aichi 467-8601, Japan.
5. Department of Respiratory Medicine and Clinical Immunology, Osaka University Graduate School of Medicine, Suita 565-0871, Japan.
6. Laboratory for Systems Genetics, RIKEN Center for Integrative Medical Sciences, Tsurumi 230-0045, Japan.
7. Department of Otorhinolaryngology-Head and Neck Surgery, Osaka University Graduate School of Medicine, Suita 565-0871, Japan.
8. Department of Genome Informatics, Graduate School of Medicine, the University of Tokyo, Tokyo 113-8654, Japan.
9. Laboratory of Children's health and Genetics, Division of Health Sciences, Osaka University Graduate School of Medicine, Suite 565-0871, Japan.
10. Department of Molecular Biology, Princeton University, Lewis Thomas Laboratory, Washington Road, Princeton, New Jersey, USA.
11. Laboratory of Statistical Immunology, Immunology Frontier Research Center (WPI-IFReC), Osaka University, Suita 565-0871, Japan.
12. Premium Research Institute for Human Metaverse Medicine (WPI-PRIMe), Osaka University, Suita 565-0871, Japan.

Contents:

- Supplemental Methods
- Supplemental References
- Supplemental Figures: 5
- Supplemental Table: 4

Supplemental Methods

Animal procedures

All experimental procedures were performed in accordance with the NIH Guide for the Care and Use of Laboratory Animals and were approved by the Animal Care and Use Committee of the Faculty of Medicine, Nagoya City University Graduate School of Medical Sciences (H30M-01). Animals were monitored daily for clinical signs, including reduced mobility, weight loss (>20%), or abnormal behavior. No animals met humane endpoints during the experiment. Male C57BL/6J wild-type mice were purchased from Charles River Japan, Inc. (Yokohama, Japan). The mice were fed standard chow (AIN-93M; Oriental Yeast Co., Tokyo, Japan) and had free access to water. Male mice aged 8 to 10 weeks were assigned to five groups (n = 4) as follows: control (no glyoxylate treatment), Day 01 (treated with glyoxylate for 1 day), Day 03 (treated with glyoxylate for 3 days), Day 06 (treated with glyoxylate for 6 days), and Day 12 (treated with glyoxylate for 6 days followed by a 6-day washout period without treatment). Glyoxylate was administered via intraabdominal injection at a dose of 100 mg/kg, as previously described[1]. Blood, 24-hour urine samples, and kidney tissues were obtained from four mice in each group. Blood and urinary biochemistry were examined using Mitsubishi Chemical Medicine (Tokyo, Japan). The urinary pH and volumes were measured manually. Kidney samples were dissected into appropriate sizes and utilized for gene expression and proteome analyses. The samples used for gene expression analysis were stored in RNAlater® (Qiagen), whereas those used for proteomic analysis were immediately frozen and stored at -80°C. To eliminate the effects of residual blood in the kidneys, PBS was perfused through the heart during sacrifice to remove the blood. This procedure was performed under sevoflurane anesthesia to minimize animal suffering.

Histology

The kidneys were fixed in 4% formalin, dehydrated, and embedded in paraffin for histological analysis. Evaluation of calcium oxalate crystals within the kidneys was conducted using Pizzolato staining. Quantification of calcium oxalate involved measuring the area of crystal deposits relative to the total kidney area using ImageJ software (version 1.53).

RNA-seq

Total RNA was extracted from kidney tissues using the RNeasy Micro Kit (Qiagen). Libraries were pooled and sequenced using the Illumina NextSeq500 platform, generating 76-base single-end reads to a depth of over 2 million reads per sample. The resulting reads were aligned to the mouse reference genome (Mus_musculus.GRCm38.dna.toplevel.fa) using Ensembl annotation (Mus_musculus.GRCm38.102. chr.gtf) using the STAR aligner (version 2.7.9a)[2]. RSEM (version 1.3.3) was used to quantify transcript abundance quantification[3]. We used the R Bioconductor package edgeR (version 3.36.0)[4] for quality control and differential expression analyses. We

removed low-expression genes and performed TMM normalization in the quality control step. Differential expression analysis involved calculating differentially expressed genes using likelihood ratio tests with the 'glmLRT' function. We used the Benjamini-Hochberg method for multiple testing correction, with a significance threshold set at 0.05. When performing comparative analysis with proteomics data, we converted the ENSEMBL IDs to MGI IDs. This conversion utilized the R Bioconductor packages org.Mm.eg.db (version 3.16.0) and AnnotationDbi (Version 1.60.0).

Peptide preparation for MS analysis

Kidney tissues were lysed with lysis buffer (10 mM Tris [pH 7.5], 250 mM sucrose, 1 mM EDTA, 2% SDS, 1× protease phosphatase inhibitor cocktail) using handy homogenizer. Equal amounts of protein were incubated with TCEP and 2-chloroacetamide for 30 min at 55°C. Resulting proteins were precipitated with methanol-chloroform. Precipitated proteins were diluted to 1 mg/mL with 25 mM HEPES-KOH (pH 8.2). MS grade trypsin (20 µg) was added to 1 mg of protein. The samples were incubated overnight at 37°C. Trifluoroacetic acid (TFA) was added to a final concentration of 1%, and the samples were incubated for 5 min at 4°C. The peptides were centrifuged at 14,000 g for 5 min at 20°C. One-tenth and nine-tenths of the resulting supernatants were used for whole proteomic and phosphoproteomic analysis, respectively, as previously described.[5] For whole proteomic analysis, peptides were desalted with styrenedivinylbenzene-reversed phase sulfonate membranes using the StageTip method[6], eluted with an elution buffer (5% ammonium hydroxide, 80% acetonitrile), dried in a MicroVac (TOMY), and dissolved in a buffer (1% formic acid, 2% acetonitrile). For phosphoproteomic analysis, the resulting peptides were concentrated to one-six volume in a MicroVac. A loading buffer (1 M glycolic acid, 70% acetonitrile, 5% TFA) was added, followed by centrifugation at 14,000 g for 5 min at 20°C. The resulting supernatants were enriched with 6.2 mg TiO2 resin for 10 min, washed with the loading buffer, followed by washing twice each with buffer 1 (80% acetonitrile, 1% TFA) and buffer 2 (20% acetonitrile, 0.2% TFA). Peptides were eluted sequentially with 1% ammonium hydroxide, followed by elution with 20% acetonitrile, 0.8% ammonium hydroxide. The elutions were combined, dried, and dissolved in a buffer (1% formic acid, 2% acetonitrile).

LC-MS/MS analysis for label free quantification

Peptides were applied to a nanoflow LC system (Dionex Ultimate 3000 nanoRSLC) equipped with an EASY-Spray ion source (C18, 25 cm × 75 µm, particle size of 2 µm). The peptides were fractionated with a linear gradient of solvent A (0.1% formic acid) and solvent B (100% acetonitrile, 0.1% formic acid) from 5% to 30% solvent B over 150 min at a flow rate of 250 nl/min. Eluted peptides were sprayed directly into a Q-Exactive HF. MS and MS/MS spectra were obtained automatically in full MS / data dependent MS2 mode. The full scan range was set to 350-1,800 m/z at 120,000 resolution, an automatic gain control (AGC) of 3e6 was used, 30 ms maximum injection

time (MIT) was allowed, and the spectra were recorded in profile. A data-dependent MS2 scan was performed on the top 20 most intense precursor ions, with a 45 s dynamic exclusion duration. The MS2 isolation windows were set to 1.2 m/z, AGC setting was 1e5, and the MIT was 150 ms. All MS/MS spectra were compared with protein sequences in the Uniprot database containing Mus musculus and common contaminants with the use of the Sequest algorithm in Proteome Discoverer v2.2. The spectral recalibration node was utilized to perform off-line recalibration of mass accuracy. A fully tryptic constraint was placed on the database search, the allowed number of missed cleavages was set at 2. Static carbamidomethylation of cysteine, dynamic acetylation of N-terminal methionine, dynamic acetylation of N-terminus with methionine loss, dynamic oxidation of methionine, and dynamic phosphorylation of serine, threonine, and tyrosine were selected. Precursor mass tolerance of 5 ppm and fragment mass tolerance of 0.02 Da were used. Reverse sequence database searches with a false discovery rate of 1% were performed. The PtmRS node and the Minora feature detector node were used with default settings to determine phosphosite localization and to detect chromatographic peaks and features for label-free MS1 quantification, respectively. A feature mapper node was used to create a consensus file in Proteome Discoverer. For phosphosite assignment confidence, a PTM-site probability threshold was set as 75%. Protein and peptide abundances were normalized by the total peptide signal detected per sample.

Proteomics data analysis

The protein or peptide abundance data obtained through LC-MS underwent quality control and differential expression analysis using the limma (version 3.54.0) package in R Bioconductor. Proteins or peptides with missing values were excluded from the analysis based on the following criteria: given the study's replication number of four per group, molecules with missing values in four or more samples were excluded. In addition, molecules with three or more missing values within each group were omitted. Following this step, the missing values were imputed using half of the minimum quantifiable value for each molecule. Subsequently, the evaluation encompassed 4,969 proteins in the whole cell proteome and 7,217 peptides in the phosphoproteome. Normalization was performed using the vsn (version 3.70.0) package in R Bioconductor. Differential expression analysis was performed using the 'lmFit' function from the limma package, with significance levels calculated using the Benjamini-Hochberg method at an FDR threshold of 0.05. To compare protein or phosphopeptide abundance quantified by proteomics with gene expression levels measured by RNA-seq, the corresponding pre-transcriptional genes of each protein were used to establish the linkage. When multiple genes could be associated with a single protein, the annotation was prioritized based on the gene IDs quantifiable by RNA-seq.

GWAS summary analysis

We analyzed publicly available GWAS summary data on urolithiasis to quantify human genetic risk

at the gene, gene set, and cell-type levels. The GWAS summary utilized for this study was a meta-analysis of urolithiasis GWAS results from a Japanese cohort (BioBank Japan) and European cohorts (UK Biobank and FinnGen)[7]. The cohorts included case and control samples as follows: 11,699 cases and 167,027 controls for the Japanese population, and 6,223 cases and 482,123 controls for the European population. Sex chromosomal regions were excluded from the analysis. To discover urolithiasis-associated genes, we used multi-marker analysis of genomic annotation (MAGMA version 1.09)[8]. The NCBI human genome build 37 location file, which contains variants and gene locations, and the 1,000 Genomes European panel were obtained from the MAGMA website and used as the annotation file and reference genome, respectively. We used a 10 kb window upstream and downstream of each gene to capture regulatory elements in our analysis. Varinat *p*-values are summarized into gene *p*-values using the MAGMA SNP-wise mean-model. Gene set enrichment analysis was subsequently performed using MAGMA based on Gene Ontology (GO) Biological Process annotations to identify biological pathways enriched for urolithiasis-associated genetic signals. Gene-level association statistics derived from MAGMA were used as input, and competitive gene set analysis was conducted to test whether genes within each GO Biological Process term were more strongly associated with urolithiasis than genes outside the gene set. When comparing the mouse data, we used orthologs to convert human gene IDs to mouse gene IDs. To assess the concentration of genetic disease risk across organs, we use FUMA (v1.3.8)[9] and LDSC (v1.0.1)[10]. For FUMA, we input the 50 genes with the lowest *p*-values obtained from MAGMA analysis, allowing us to evaluate their expression levels across 54 tissue types defined in GTEx v8. Additionally, we assessed the concentration of genetic risk in specific chromosomal regions across organs using partitioned LDSC[11] on the GWAS Summary data. The 1000 Genomes Project European reference panel was used for the reference genome. We obtained LD score files for each organ-specific region on the chromosomes and baseline model LD score files from the download site (https://alkesgroup.broadinstitute.org/LDSCORE/) of the Alkes Group, the developers of LDSC. To further evaluate the enrichment of urolithiasis genetic risk at the kidney cell-type level, we applied stratified LD score regression specifically expressed genes (LDSC-SEG). Cell-type-specific genes were defined using RNA-seq data from the Kidney Precision Medicine Project (KPMP). For each cell type, pseudo-bulk expression profiles were calculated, and genes with a tau score ≥ 0.8 were designated as cell-type-specific. Consistent with the analyses described above, the European 1000 Genomes Project genotype data were used as the reference panel. Next, we used scDRS (v1.0.0)[12] to evaluate the genetic risk concentration in various cell types within a mouse kidney single-cell RNA-seq dataset and KPMP dataset for urolithiasis GWAS. Initially, we defined a set of 1000 genetically disease-related genes by sorting the genes in ascending order of their *p*-values obtained from the MAGMA results. Subsequently, scDRS performed 1000 Monte Carlo control score estimations using a random gene set and calculated disease scores for each cell in the single-cell RNA-seq dataset by assessing the

expression of disease-related genes, resulting in empirical *p*-values for the disease relevance of each cell, which is defineed disease relevant score (DRS). The ‘score_cell’ function was used with default parameters, and Z-scores computed using MAGMA were used for weighting. Finally, scDRS assessed the cell types where genetic risk from GWAS was concentrated using the ‘downstream_group_analysis’ function with default settings and reported the results in terms of *p*-values. In particular, DRS were aggregated by cell type, and group-level statistics were computed as the mean DRS across cells within each cell type. To assess significance, the observed group-level statistic was compared against a null distribution reconstructed with consideration of the number of cells per group, using Monte Carlo simulations based on matched control gene sets. Empirical *p*-values were then calculated as the proportion of null statistics greater than or equal to the observed value.

Gene set enrichment analysis

Gene set enrichment analysis was conducted to interpret the biological implications of differentially expressed molecules obtained from RNA-seq, whole-cell proteome, and phosphoproteome analyses. The 'enricher' function from the ‘clusterprofiler’ package (version 4.6.0) in R Bioconductor was employed for hypergeometric analysis. For interpreting RNA-seq and whole-cell proteome results, Gene Ontology (GO) terms from the Biological Process category were used as gene sets for enrichment analysis. Because functionally similar or redundant GO terms are often simultaneously detected, semantic similarity–based simplification was applied to reduce redundancy among enriched gene sets. This simplification was performed using the ‘simplify’ function in clusterProfiler, which calculates semantic similarity between GO terms and retains representative terms. For phosphoproteome data, enrichment analysis was used to identify upstream kinases that may be responsible for the observed phosphorylation changes. Kinase–substrate relationships, including kinase names and corresponding phosphorylation sites, were obtained from the PhosphoSitePlus[13] (https://www.phosphosite.org/homeAction.action) database and used to construct kinase-based gene sets. Kinase set enrichment analysis was then performed using these curated gene sets to infer kinase activities associated with differential phosphorylation.

Clustering

Clustering of RNA-seq, whole cell proteome, and phosphoproteome expression profiles for each molecule was calculated using the 1-Pearson's correlation coefficient as the distance between molecules. The clustering itself employed the ‘hclust’ function, which is a built-in R function, using the average method. For the integrated analysis between human GWAS data and the hyperoxaluric mouse model data, hierarchical clustering was performed using Euclidean distance as the distance metric. For followu-up analysis, distances between molecules were calculated using Pearson's correlation coefficient. The clustering outcomes were visualized using the

ComplexHeatmap package (version 2.14.0) available in R Bioconductor.

Processing of single-cell RNA-seq reference datasets

We analyzed publicly available single-cell RNA-seq data from mouse[14] and human kidneys to determine which cell types are anatomically essential for urolithiasis formation. The Susztak laboratory generated the mouse dataset, and the human dataset was produced by the Kidney Precision Medicine Project (KPMP). About mouse data, we specifically extracted cells from adult mice for analysis, as the dataset contained cells from both neonatal and adult mice. The cell types defined in the original data were as follows: DCT, Early_PT, Endo, IC, LOH, Macro, NP, Neutro, PC, PCT, PST, Podo, Proliferating, Stroma_1, Stroma_2, and Unnamed. We reclassified these based on the results of UMAP clustering performed exclusively on adult mouse cells. PST, PCT, and NP were classified as Proximal representing proximal tubular cells; DCT and LOH were classified as Distal representing distal tubular cells; Neutro and Macro were categorized as Immune representing immune cells; PC was designated as PC, representing the collecting duct principal cells; IC was designated as IC, representing the collecting duct intercalated cells; Stroma_1 and Stroma_2 were categorized as Stroma, representing renal stromal cells. Early_PT and Proliferating cells were excluded from the analysis because they have intermediate gene expression profiles between Proximal and Distal, which would interfere with identifying cell types essential for urinary stone formation in this study. Additionally, Endo, Podo, and Unnamed cell types were excluded from the analysis because they had fewer than 200 cells. For the human single-cell RNA-seq dataset from the Kidney Precision Medicine Project (KPMP), we restricted the analysis to cells obtained from living donor samples, since the original dataset contained both living donor and disease-derived cells. We then redefined the cell types as follows: Proximal, comprising PT-S1/S2, PT-S3, and dPT; Distal, comprising DTL1, dC-TAL, C-TAL, M-TAL, aTAL1, aTAL2, DCT1, dDCT, CNT, and dCNT; PC, comprising PC, CNT-PC, dCNT-PC, dPC, and tentatively tPC-IC; IC, comprising IC-A, IC-B, CNT-IC-A, and dIC-A; Immune, comprising T-REG, T-CYT, T, cycT, NKT, NK1, NK2, B, PL, MON, ncMON, cycMNP, MAC-M2, MAST, MDC, cDC, and pDC; and Stroma, comprising FIB, aFIB, MyoF, VSMC, dVSMC, VSMC/P, and MC. Other minor cell types, including POD, REN, and cycEPI, were grouped into the "Other" category. To identify cell type–specific genes, we generated pseudo-bulk expression profiles for both the human and mouse single-cell datasets. Pseudo-bulk expression was calculated using the ‘AverageExpression’ function in Seurat (version 5.3.0)[15]. For each gene, we computed the tau score, and genes with a tau score ≥ 0.8 were defined as cell–type–specific.

Integration of human and mouse data

To integrate human and mouse data, human gene IDs were converted to mouse gene IDs using an ortholog reference. Ortholog reference data were obtained from Mouse Gene Informatics

(https://www.informatics.jax.org/downloads/reports/HOM_MouseHumanSequence.rpt)[16]. Among the 40,649 genes evaluated in the MAGMA analysis, 18,687 could be successfully converted to mouse gene IDs. Of the 17,249 converted genes, 16,622 (96.4%) were protein-coding, whereas only 1,011 (3.6%) of the non-converted genes were protein-coding, with the vast majority corresponding to pseudogenes or non-coding RNAs. There were 1,392 human genes for which a one-to-one conversion to mouse gene IDs could not be established. The whole correspondence table between human and mouse genes is provided in **Supplementary_data1.xlsx**. Subsequently, we evaluated the *p*-values from the mouse analysis and MAGMA-based *p*-values from the human GWAS. The number of molecules that were concurrently significant in both studies was assessed using Fisher's exact test. The threshold for significance in this assessment was set at an FDR value < 0.05.

Cell type deconvolution analysis

Cell type deconvolution was performed using CIBERSORTx[17] (https://cibersortx.stanford.edu/) to estimate relative cell-type composition in bulk RNA-seq samples from the hyperoxaluric mouse model. The analysis proceeded in two main steps: (1) construction of a cell-type signature matrix from single-cell RNA-seq reference data, and (2) deconvolution of bulk RNA-seq samples to obtain estimated cell fractions using the CIBERSORTx *Cell Fractions* module. For input data preparation, gene-level expression for all bulk RNA-seq samples from the hyperoxaluric mouse model was normalized to transcripts per million (TPM). Genes with low expression across samples (mean TPM < 1) were removed to reduce noise. The mouse single-cell RNA-seq reference dataset was used to define cell types and generate signatures. Cells of single-cell RNA-seq data were assigned to biologically meaningful cell-type labels before signature construction. Only genes common to both the bulk and single-cell matrices were retained for downstream analysis. A signature matrix was generated from the annotated single-cell reference using CIBERSORTx’s signature creation workflow (single-cell mode). To account for technical differences between single-cell and bulk RNA-seq platforms, we enabled CIBERSORTx’s single-cell adjustment procedures (S-mode) and applied batch-correction options where available. The *Cell Fractions* module was then run using the created signature matrix to deconvolute each bulk sample into relative cell-type proportions. Key runtime parameters were: quantile normalization disabled, 100 permutations for significance testing. CIBERSORTx implements a support vector regression (ν-SVR)-based algorithm to solve the deconvolution problem and uses permutations to provide empirical p-values and confidence in the estimated fractions. The output from CIBERSORTx consisted of a matrix of estimated cell-type fractions for each bulk sample together with associated p-values and confidence intervals. These estimated fractions were subsequently used to evaluate differences in cell composition between experimental groups and to interpret modality-specific differential expression results.

Randall's plaque microarray analysis

Gene expression analysis of Randall's plaque, the stone-forming site in urolithiasis patients, was conducted using microarray data obtained from the GEO repository (GSE73680)[18]. Since this dataset included information on urolithiasis patients and healthy controls, only data from patients with urolithiasis were utilized. Differential expression genes were assessed using the limma package (version 3.54.0) in R Bioconductor, using the 'lmFit' function and incorporating age as a covariate. Quality control was conducted using limma. Sample-wise correlation coefficients and principal component analyses were used to assess data. Samples with genetic profiles different from those of other samples were excluded from the analysis. The 'subtract' method within the 'backgroundCorrect' function was utilized for background correction, and the 'quantile' method within the 'normalizeBetweenArrays' function was applied for normalization. Samples exhibiting markedly aberrant gene expression profiles were excluded. A significance threshold of adjusted FDR (Benjamini-Hochberg method) < 0.05 was applied to the evaluated genes.

Permutation analysis

A permutation analysis was performed using shuffling of gene labels to validate the findings from Randall's plaque microarray analysis and demonstrate that the results were not obtained by chance. Among the 46 cross-species urolithiasis-related molecules, 42 genes were quantifiable in the human renal papillae gene expression data. 8,968 genes were quantifiable in this dataset, assessed by GWAS analysis using MAGMA, and included in at least one mouse kidney multi-omics analysis modality. From these 8,968 genes, 42 were randomly selected 20,000 times to evaluate the number of genes with $P < 0.05$ and those with FDR values < 0.05 in the differential expression analysis in renal papillae data. Given the anatomical overlap of renal papilla specimens with parts of the collecting duct and Henle's loop, we focused on molecules derived from collecting duct principal cells or proximal tubular cells. We counted the number of genes with $P < 0.05$ or FDR < 0.05 derived from collecting duct principal cells or proximal tubular cells and used these counts to construct a null distribution for the analysis.

Colocalization analysis

We conducted a colocalization analysis to assess whether effective varinats shared between urolithiasis GWAS and eQTL summary statistics were present within the target gene loci. We filtered GWAS summary statistics within a range of ±20kb around the upstream and downstream regions of the target gene loci for analysis. Summary statistics for kidney eQTL were obtained from NephQTL2 (https://hugeampkpn.org/research.html?pageid=nephqtl2_home_89), an eQTL atlas evaluating gene expression from microdissected glomerular and tubular regions[19]. In this analysis, we utilized both sets of summary statistics for the colocalization analysis. The 'coloc.abf' function in coloc (version 5.2.3) was used for analysis, computing the posterior probabilities of four

hypotheses: H1 (no GWAS showing effects), H2 (effects present in one GWAS only), H3 (effects present in both GWAS without colocalization), and H4 (effects present in both GWAS with colocalization)[20]. An H4 posterior probability of 75% or higher was defined as indicative of colocalization.

Mendelian randomization analysis

To investigate whether kidney eQTLs exert a causal effect on urolithiasis, we performed Mendelian randomization (MR) analysis using the TwoSampleMR[21] package in R. Variants reaching genome-wide significance ($P \leq 5.0 \times 10^{-8}$) in the eQTL dataset were selected as instrumental variables. Linkage disequilibrium (LD) clumping of candidate variants was conducted using the 1000 Genomes Project European population as the reference panel. LD-based clumping of variants was performed using PLINK 1.9[22] with the parameters --clump-r2 0.01 and --clump-kb 10000. When only a single variant was available for a given exposure, causal estimates were calculated using the Wald ratio method. In cases where multiple independent variants were available, we applied the inverse variance weighted (IVW) method to obtain pooled causal effect estimates.

1. Okada A, Nomura S, Higashibata Y, Hirose M, Gao B, Yoshimura M, et al.: Successful formation of calcium oxalate crystal deposition in mouse kidney by intraabdominal glyoxylate injection. *Urol. Res.* 35: 89–99, 2007

2. Dobin A, Davis CA, Schlesinger F, Drenkow J, Zaleski C, Jha S, et al.: STAR: ultrafast universal RNA-seq aligner. *Bioinformatics* 29: 15–21, 2013

3. Li B, Dewey CN: RSEM: accurate transcript quantification from RNA-Seq data with or without a reference genome. *BMC Bioinformatics* 12: 323, 2011

4. Robinson MD, McCarthy DJ, Smyth GK: edgeR: a Bioconductor package for differential expression analysis of digital gene expression data. *Bioinforma. Oxf. Engl.* 26: 139–140, 2010

5. Hashimoto Y, Greco TM, Cristea IM: Contribution of Mass Spectrometry-Based Proteomics to Discoveries in Developmental Biology. *Adv. Exp. Med. Biol.* 1140: 143–154, 2019

6. Rappsilber J, Mann M, Ishihama Y: Protocol for micro-purification, enrichment, pre-fractionation and storage of peptides for proteomics using StageTips. *Nat. Protoc.* 2: 1896–1906, 2007

7. Sakaue S, Kanai M, Tanigawa Y, Karjalainen J, Kurki M, Koshiba S, et al.: A cross-population atlas of genetic associations for 220 human phenotypes. *Nat. Genet.* 53: 1415–1424, 2021

8. de Leeuw CA, Mooij JM, Heskes T, Posthuma D: MAGMA: generalized gene-set analysis of

GWAS data. *PLoS Comput. Biol.* 11: e1004219, 2015

9. Watanabe K, Taskesen E, van Bochoven A, Posthuma D: Functional mapping and annotation of genetic associations with FUMA. *Nat. Commun.* 8: 1826, 2017

10. Bulik-Sullivan BK, Loh P-R, Finucane HK, Ripke S, Yang J, Schizophrenia Working Group of the Psychiatric Genomics Consortium, et al.: LD Score regression distinguishes confounding from polygenicity in genome-wide association studies. *Nat. Genet.* 47: 291–295, 2015

11. Finucane HK, Bulik-Sullivan B, Gusev A, Trynka G, Reshef Y, Loh P-R, et al.: Partitioning heritability by functional annotation using genome-wide association summary statistics. *Nat. Genet.* 47: 1228–1235, 2015

12. Zhang MJ, Hou K, Dey KK, Sakaue S, Jagadeesh KA, Weinand K, et al.: Polygenic enrichment distinguishes disease associations of individual cells in single-cell RNA-seq data. *Nat. Genet.* 54: 1572–1580, 2022

13. Hornbeck PV, Zhang B, Murray B, Kornhauser JM, Latham V, Skrzypek E: PhosphoSitePlus, 2014: mutations, PTMs and recalibrations. *Nucleic Acids Res.* 43: D512-520, 2015

14. Miao Z, Balzer MS, Ma Z, Liu H, Wu J, Shrestha R, et al.: Single cell regulatory landscape of the mouse kidney highlights cellular differentiation programs and disease targets. *Nat. Commun.* 12: 2277, 2021

15. Hao Y, Stuart T, Kowalski MH, Choudhary S, Hoffman P, Hartman A, et al.: Dictionary learning for integrative, multimodal and scalable single-cell analysis. *Nat. Biotechnol.* 42: 293–304, 2024

16. Baldarelli RM, Smith CL, Ringwald M, Richardson JE, Bult CJ, Mouse Genome Informatics Group: Mouse Genome Informatics: an integrated knowledgebase system for the laboratory mouse. *Genetics* 227: iyae031, 2024

17. Newman AM, Steen CB, Liu CL, Gentles AJ, Chaudhuri AA, Scherer F, et al.: Determining cell-type abundance and expression from bulk tissues with digital cytometry. *Nat. Biotechnol.* 37: 773–782, 2019

18. Taguchi K, Hamamoto S, Okada A, Unno R, Kamisawa H, Naiki T, et al.: Genome-Wide Gene Expression Profiling of Randall's Plaques in Calcium Oxalate Stone Formers. *J. Am. Soc. Nephrol. JASN* 28: 333–347, 2017

19. Han SK, McNulty MT, Benway CJ, Wen P, Greenberg A, Onuchic-Whitford AC, et al.: Mapping genomic regulation of kidney disease and traits through high-resolution and interpretable eQTLs. *Nat. Commun.* 14: 2229, 2023

20. Wallace C: Statistical testing of shared genetic control for potentially related traits. *Genet. Epidemiol.* 37: 802–813, 2013

21. Hemani G, Zheng J, Elsworth B, Wade KH, Haberland V, Baird D, et al.: The MR-Base platform supports systematic causal inference across the human phenome. *eLife* 7: e34408, 2018

22. Purcell S, Neale B, Todd-Brown K, Thomas L, Ferreira MAR, Bender D, et al.: PLINK: a tool set for whole-genome association and population-based linkage analyses. *Am. J. Hum. Genet.* 81: 559–575, 2007

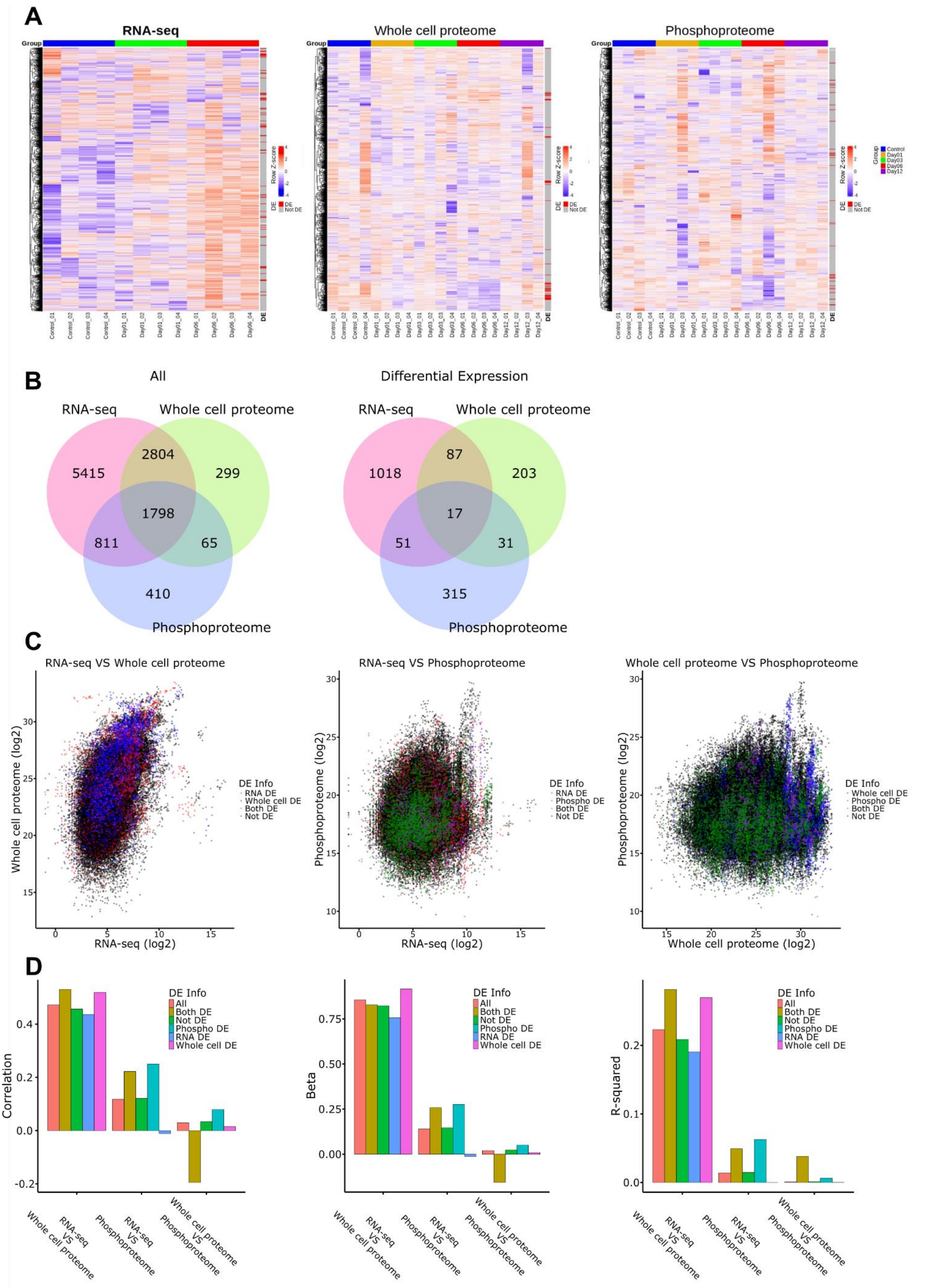
A
RNA-seq
Whole cell proteome
Phosphoproteome
Group
Row Z-score
DE
Not DE
Control
Day01
Day03
Day06
Day12
B
All
Differential Expression
RNA-seq
Whole cell proteome
Phosphoproteome
5415
2804
299
1798
811
65
410
1018
87
203
17
51
31
315
C
RNA-seq VS Whole cell proteome
RNA-seq VS Phosphoproteome
Whole cell proteome VS Phosphoproteome
Whole cell proteome (log2)
RNA-seq (log2)
Phosphoproteome (log2)
DE Info
RNA DE
Whole cell DE
Phospho DE
Both DE
Not DE
D
Correlation
Beta
R-squared
All
Both DE
Not DE
Phospho DE
RNA DE
Whole cell DE
RNA-seq VS Whole cell proteome
RNA-seq VS Phosphoproteome
Whole cell proteome VS Phosphoproteome

Supplemental Figure 1. Multi-omics analysis of the kidney from the hyperoxaluric mouse model using the three modalities.

A Heatmaps represent the expression profiles of all molecules in the RNA-seq (left), whole cell proteome (middle), and phosphoproteome (right). **B** Venn diagrams depicting the overlap of molecular structures across different modalities. In the context of proteomics data, molecules are considered pre-translated genes. The Venn diagram on the left represents the set of all molecules, whereas that on the right displays only the differentially expressed molecules. **C** Expression profiles of molecules derived from the same genes across each modality. RNA-seq data were described by log fold change of read counts, and proteome data were described by log fold change of abundance. In the plot, the red and blue points represent differential expression in one modality, the purple points represent differential expression in both modalities, and the gray points represent molecules that are not differentially expressed in either modality. (Left) RNA-seq on the x-axis and whole-cell proteome on the y-axis. (Middle) RNA-seq on the x-axis and phosphoproteome on the y-axis. (Right) Whole cell proteome on the x-axis and phosphoproteome on the y-axis. **D** The relationship between expression profiles in each modality was quantified using a correlation coefficient (right), regression coefficients of linear regression (middle), and R-square of linear regression (left). This categorizes and displays all molecules, non-differentially expressed molecules, molecules differentially expressed in one modality, and molecules differentially expressed in both modalities for the two modalities being compared.

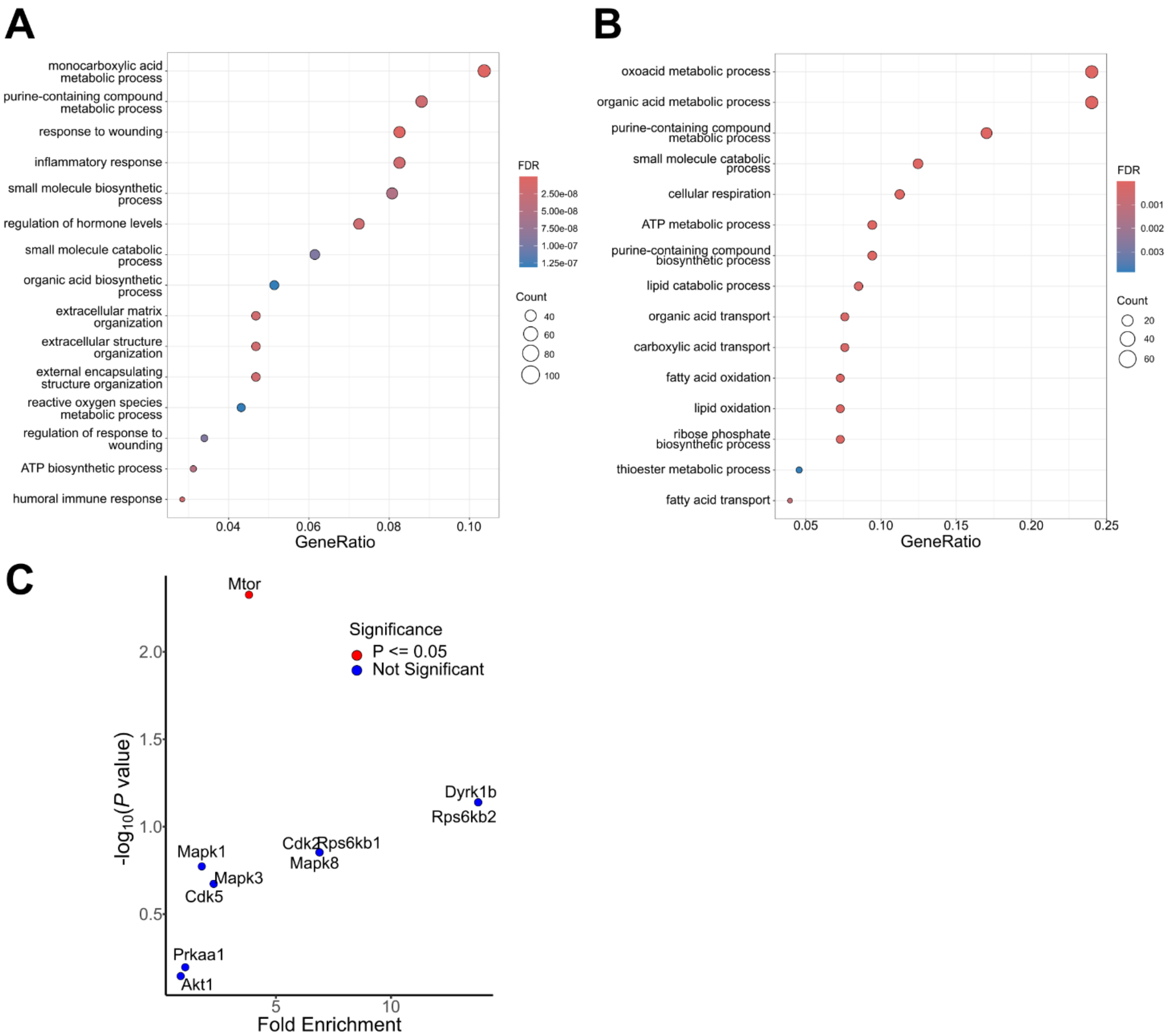

Supplemental Figure 2. Gene set enrichment analysis of the hyperoxaluric model mice.

A Gene set enrichment analysis of differentially expressed genes identified by mouse kidney RNA-seq. **B** Gene set enrichment analysis of differentially expressed proteins identified by whole-cell proteome analysis. In both panels, enrichment analysis was performed using Gene Ontology Biological Process (GO BP) gene sets. Dot size represents the number of differentially expressed molecules assigned to each GO term (“Count”), while dot color indicates the false discovery rate (FDR). The x-axis shows the GeneRatio, defined as the proportion of differentially expressed molecules relative to the total number of genes annotated to each GO term. **C** To identify which phosphorylation sites downstream of kinase activity were most affected among the detected phosphopeptides, we performed kinase set enrichment analysis. The x-axis represents the fold enrichment (odds ratio, OR), and the y-axis represents the $-\log_{10}$ *p*-value.

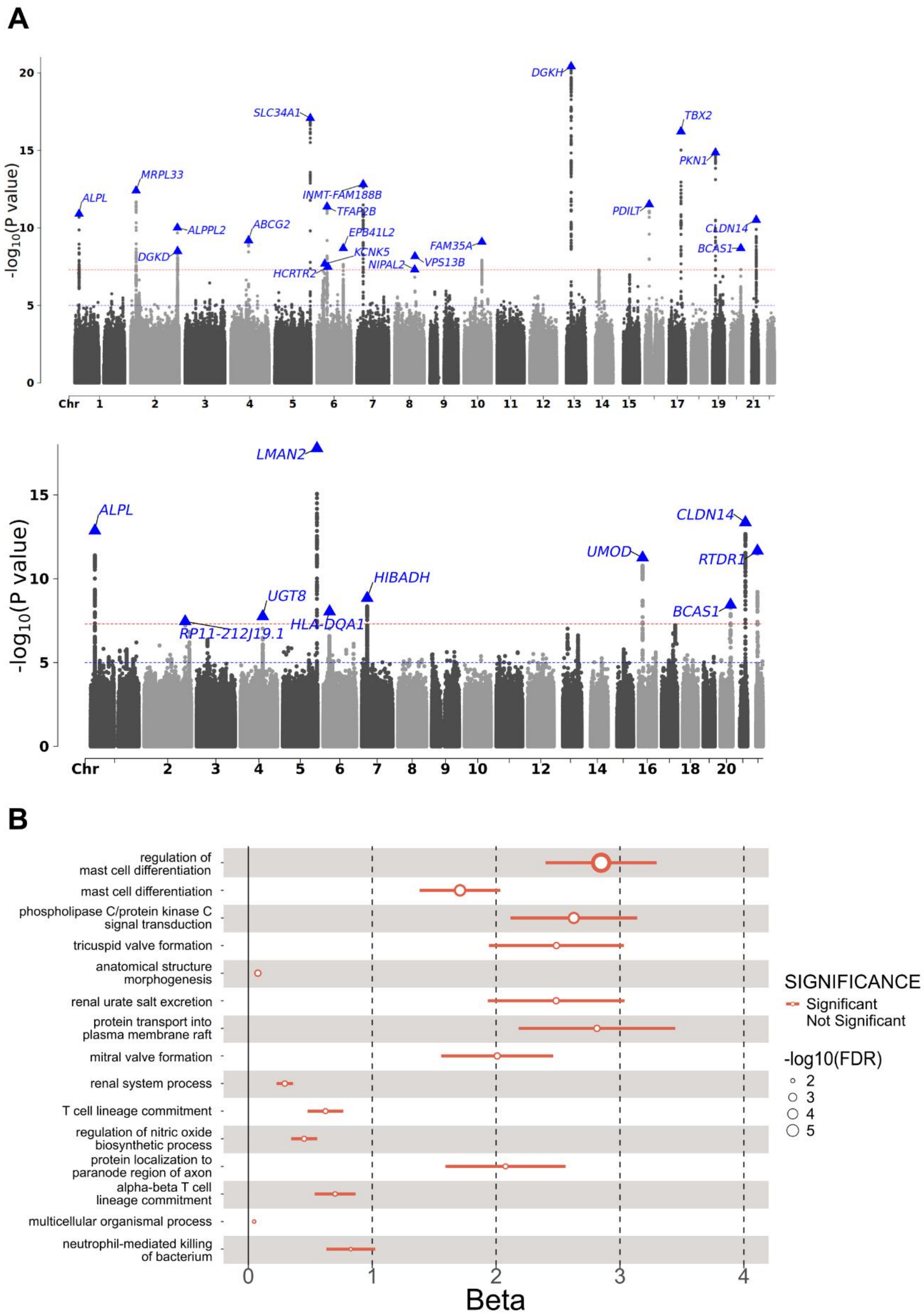

Supplemental Figure 3. Analysis of urolithiasis GWAS summary statistics.

A Manhattan plot derived from genome-wide association study (GWAS) summary statistics for urolithiasis in the Japanese population from BioBank Japan (upper) and in European populations from the UK Biobank and FinnGen (lower). **B** Gene set enrichment analysis for Gene Ontology (GO) Biological Process terms was

performed using MAGMA, based on the human urolithiasis genome-wide association study (GWAS). The forest plot displays the top 15 GO terms ranked by P value (y-axis). The x-axis represents the effect size (Beta) for each gene set, with error bars indicating the standard error (SE). Point sizes correspond to −log10-transformed false discovery rate (FDR) values. All displayed GO terms remained statistically significant after multiple testing correction (FDR < 0.05).

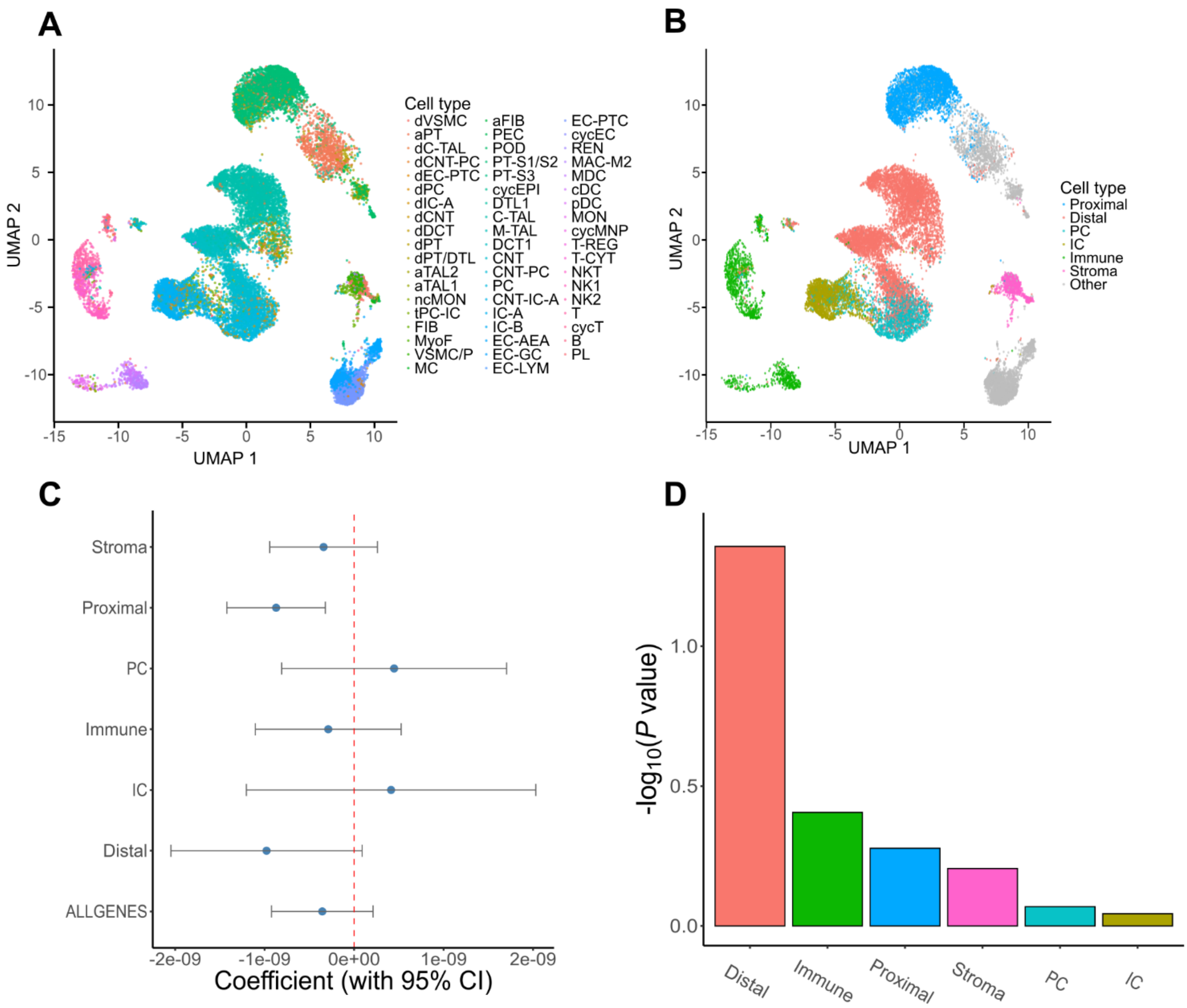

Supplemental Figure 4. Cell-type annotation and enrichment analysis of human kidney scRNA-seq data in urolithiasis GWAS.

A UMAP plot of kidney single-cell RNA-seq data obtained from the Kidney Precision Medicine Project (KPMP). Cells derived from living donors were extracted from the original dataset, which also included disease samples. The annotation shown here corresponds to the original KPMP classification. **B** UMAP plot with redefined annotation used in this study. Original cell types were consolidated into six major categories: Proximal, Distal, Principal cell (PC), Intercalated cell (IC), Immune, and Stroma. Minor cell types, including POD, REN, and cycEPI, were grouped into the "Other" category. **C** Cell type–specific enrichment analysis using LDSC-SEG. Pseudo-bulk gene expression was calculated for each redefined cell type, and cell–type–specific genes were identified based on expression specificity. LDSC-SEG was then performed to evaluate enrichment of urolithiasis GWAS signals. The category ALLGENES represents an analysis including all protein-coding genes, used as a baseline reference for comparison with individual cell types. **D** Cell type enrichment analysis using scDRS applied directly to the kidney single-cell RNA-seq data. The bar plot illustrates the degree of enrichment (in terms of -log10 *p*-values) of genetic risk for urolithiasis across redefined kidney cell types. No cell type showed statistically significant enrichment.

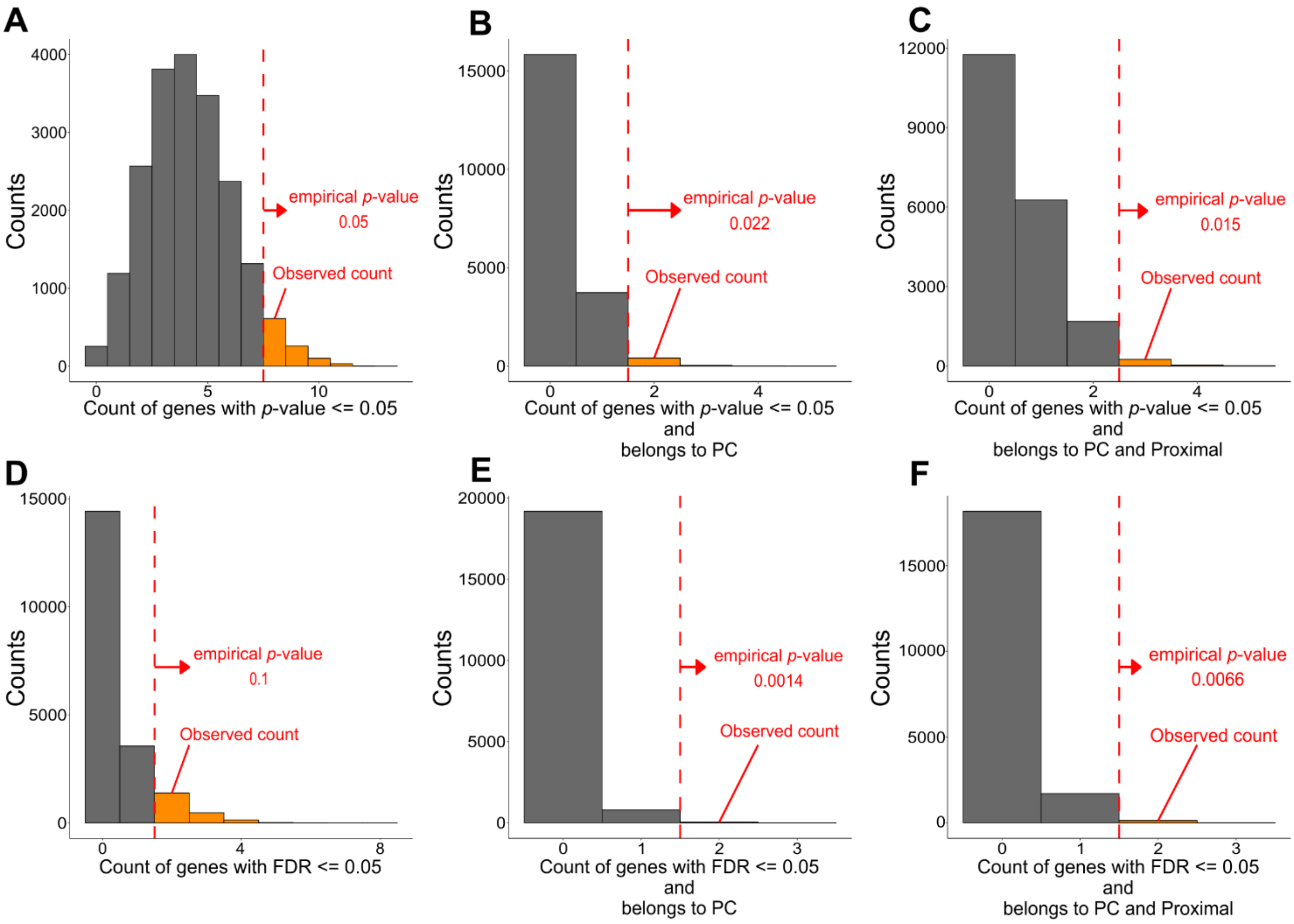

Supplemental Figure 5. Follow-up analysis of human renal papilla gene expression data using permutation Tests

A In the validation analysis, which verified whether 46 urolithiasis-related candidate molecules obtained by cross-species analysis were expressed in the presence of urinary stones in the human renal papillae, a permutation test was performed to show that the results were not coincidental. Therefore, 42 genes were randomly selected from the gene expression data of the human renal papillae, and the null distribution was obtained by repeating 20,000 times to determine the number of molecules with a $p \leq 0.05$. The empirical *p*-value for obtaining more than the observed number of molecules with a *p*-value of the differential expression analysis ≤ 0.05 was 0.044. **B** To further verify the anatomical validity, an additional permutation test was conducted under the condition that the molecules had a $p \leq 0.05$ and were specifically expressed in the collecting duct principal cells. The empirical *p*-value for this test was 0.021. **C** Considering that the renal papilla includes tissue from the loop of Henle, which is classified as a proximal tubule in the single-cell RNA-seq data used in this study, the same permutation analysis was performed under the condition that the molecules had a $p \leq 0.05$ and were specifically expressed in the collecting duct principal cells or proximal tubules. The empirical *p*-value for this test was 0.014. **D** The permutation test was repeated, altering the criterion from a *p*-value of ≤ 0.05 to an FDR ≤ 0.05. The empirical *p*-value was 0.1. **E** The empirical *p*-value for being specific to collecting duct principal cells with an FDR value ≤ 0.05 was 0.0012. **F** The empirical *p*-value for being specific to collecting duct principal cells or proximal tubules with an FDR value ≤ 0.05 was 0.0064.

Supplemental Table 1. Experimental data for the hyperoxaluric mouse model.

	GROUP					
	Control	Day01	Day03	Day06	Day12	*p* value
Serum						
Creatinine, mg/dl	0.07 (0.01)	0.09 (0.05)	0.08 (0.02)	0.10 (0.02)	0.10 (0.04)	0.301
Blood urea nitrogen, mg/dl	12.68 (2.54)	17.00 (4.15)	17.08 (1.53)	27.38 (5.19)	16.68 (7.31)	**0.043**
Ca, mg/dl	2.12 (0.54)	2.20 (0.28)	2.52 (0.46)	3.08 (1.33)	4.47 (3.21)	0.466
P, mg/dl	20.95 (0.48)	19.8 (0.26)	20.45 (0.79)	20.32 (2.69)	16.48 (5.50)	0.138
Na, mEq/l	134.75 (5.68)	131.50 (2.38)	136.25 (5.85)	144.25 (5.62)	140.75 (6.24)	**0.031**
Mg, mg/dl	0.75 (0.17)	0.68 (0.10)	0.82 (0.15)	1.00 (0.39)	1.52 (1.16)	0.521
K, mEq/l	4.62 (0.56)	4.45 (0.31)	4.90 (0.42)	4.65 (0.26)	4.78 (0.85)	0.578
Cl, mEq/l	113.00 (4.16)	110.00 (1.63)	114.25 (5.32)	119.75 (2.99)	111.50 (2.08)	**0.049**
Urine						
Volume, ml	1.00 [0.95, 1.02]	1.25 [0.48, 2.15]	1.65 [1.60, 1.92]	1.00 [0.65, 1.27]	1.15 [0.85, 1.53]	0.248
pH	6.00 [6.00, 6.25]	6.00 [6.00, 6.25]	6.00 [6.00, 6.25]	8.00 [7.75, 8.00]	5.50 [5.00, 6.50]	0.074
Ca, μmol/g/Cr	10.35 [8.60, 10.77]	2.20 [1.55, 2.83]	2.40 [2.03, 2.72]	2.65 [2.15, 3.12]	8.95 [7.70, 11.23]	**0.007**
P, μmol/g/Cr	47.85 [45.38, 59.50]	41.10 [40.58, 42.18]	42.40 [39.10, 44.10]	47.65 [46.55, 48.40]	46.70 [45.45, 48.35]	0.051
Na, μmol/g/Cr	149.50 [140.50, 153.25]	74.50 [53.75, 100.00]	122.50 [101.00, 136.75]	64.50 [44.00, 78.75]	102.00 [82.75, 130.25]	**0.039**
Mg, μmol/g/Cr	87.10 [73.15, 90.35]	22.70 [11.60, 36.10]	33.05 [22.87, 35.85]	1.50 [0.60, 2.52]	34.30 [22.17, 52.30]	**0.049**
K, μmol/g/Cr	305.80 [253.83, 328.38]	122.20 [93.93, 163.43]	119.45 [103.12, 132.38]	107.95 [103.38, 121.03]	142.10 [102.05, 240.75]	0.241
Cl, μmol/g/Cr	314.00 [244.00, 360.50]	125.00 [104.00, 144.25]	124.50 [114.00, 139.75]	67.00 [45.25, 99.00]	101.50 [55.92, 180.75]	0.119
Oxalate, μmol/g/Cr	13.89 [11.76, 17.92]	7.83 [5.94, 10.18]	12.46 [9.64, 15.89]	27.39 [26.87, 32.23]	12.36 [11.35, 13.26]	**0.026**
Adherent renal crystals						
Ratio of crystal area, ‰	0.012 [0.0052, 0.026]	0.014 [0.56, 0.21]	0.0017 [0, 0.0059]	6.80 [5.43, 8.28]	0.0073 [0.0045, 0.011]	**0.009**

Ca, calcium; P, phosphorus; Na, sodium; Mg, magnesium; K, potassium; Cl, Chloride; Cr, creatinine.; COX, calcium oxalate.

Values are presented as mean (SD), or median [interquartile range].

Supplemental Table 2. Human–mouse orthologs of cross-species urolithiasis-associated candidate molecules.

Human Gene Symbol	Human ENTREZ Gene ID	Human Gene Position	Mouse ENSEMBL Gene ID	Mouse UNIPROT ID
ABCG2	9429	chr4:89,001,416-89,162,778	ENSMUSG00000029802	Q7TMS5
APOM	55937	chr6:31,610,181-31,635,987	ENSMUSG00000024391	Q9Z1R3
BAG6	7917	chr6:31,596,805-31,630,438	ENSMUSG00000024392	Q9Z1R2
C4A	720	chr6:31,939,834-31,980,458	ENSMUSG00000073418	P01029
CASR	846	chr3:121,892,515-122,020,476	ENSMUSG00000051980	Q9QY96
CDK12	51755	chr17:37,607,739-37,700,797	ENSMUSG00000003119	Q14AX6
CLDN10	9071	chr13:96,076,009-96,242,013	ENSMUSG00000022132	Q9Z0S6
CRYAB	1410	chr11:111,769,288-111,804,464	ENSMUSG00000032060	P23927
DDAH2	23564	chr6:31,684,817-31,708,040	ENSMUSG00000007039	Q99LD8
EPB41L3	23136	chr18:5,382,385-5,640,662	ENSMUSG00000024044	Q9WV92
FADS2	9415	chr11:61,573,675-61,644,826	ENSMUSG00000024665	Q9Z0R9
FBN1	2200	chr15:48,690,510-48,947,906	ENSMUSG00000027204	Q61554
GLUD1	2746	chr10:88,799,959-88,864,600	ENSMUSG00000021794	P26443
H1-0	3005	chr22:38,191,236-38,213,439	ENSMUSG00000096210	P10922
HIBADH	11112	chr7:27,555,061-27,712,502	ENSMUSG00000029776	Q99L13
HLA-B	3106	chr6:31,311,652-31,334,956	ENSMUSG00000061232	P01899
HLA-DQA1	3117	chr6:32,595,183-32,621,461	ENSMUSG00000036594	P14434
HLA-DRB1	3123	chr6:32,536,552-32,567,625	ENSMUSG00000060586	O78196
HMGCS1	3157	chr5:43,277,572-43,323,514	ENSMUSG00000093930	Q8JZK9
HSPA12A	259217	chr10:118,420,703-118,619,233	ENSMUSG00000025092	Q8K0U4
HSPA1B	3304	chr6:31,785,515-31,808,031	ENSMUSG00000090877	Q61696
LUZP1	7798	chr1:23,400,523-23,505,349	ENSMUSG00000001089	Q8R4U7
MAPT	4137	chr17:43,961,920-44,115,700	ENSMUSG00000018411	P10637
MXD3	83463	chr5:176,722,500-176,748,899	ENSMUSG00000021485	Q80US8
NCCRP1	342897	chr19:39,677,604-39,702,524	ENSMUSG00000047586	G3X9C2
NDUFAB1	4706	chr16:23,582,335-23,617,637	ENSMUSG00000030869	Q9CR21
PLCB1	23236	chr20:8,102,913-8,875,547	ENSMUSG00000051177	Q9Z1B3
PLCL1	5334	chr2:198,659,317-199,024,587	ENSMUSG00000038349	Q3USB7
PPHLN1	51535	chr12:42,709,969-42,852,423	ENSMUSG00000036167	Q8K2H1
PRRC2A	7916	chr6:31,578,492-31,615,548	ENSMUSG00000024393	Q7TSC1
RAP1GAP	5909	chr1:21,912,714-22,005,850	ENSMUSG00000041351	A2ALS5
SHROOM3	57619	chr4:77,346,382-77,714,406	ENSMUSG00000029381	Q9QXN0
SLC12A3	6559	chr16:56,889,119-56,959,762	ENSMUSG00000031766	P59158
SLC20A1	6574	chr2:113,393,516-113,431,402	ENSMUSG00000027397	Q61609
SLC25A21	89874	chr14:37,137,126-37,651,811	ENSMUSG00000035472	Q8BZ09
SLC34A1	6569	chr5:176,801,435-176,835,849	ENSMUSG00000021490	Q9D2V6
SLCO4C1	353189	chr5:101,559,690-101,641,988	ENSMUSG00000040693	Q8BGD4
SNX17	9784	chr2:27,583,483-27,610,402	ENSMUSG00000029146	Q8BVL3
STAB1	23166	chr3:52,519,354-52,568,511	ENSMUSG00000042286	G3X973
TCEA3	6920	chr1:23,697,402-23,761,241	ENSMUSG00000001604	P23881
TMEM252	169693	chr9:71,141,496-71,165,799	ENSMUSG00000048572	Q8C353
TRPM7	54822	chr15:50,839,355-50,988,994	ENSMUSG00000027365	Q923J1
TRPS1	7227	chr8:116,410,724-116,691,202	ENSMUSG00000038679	Q3UQJ9
UGT8	7368	chr4:115,509,558-115,609,381	ENSMUSG00000032854	Q64676
UMOD	7369	chr16:20,334,373-20,374,037	ENSMUSG00000030963	Q91X17
VCL	7414	chr10:75,747,874-75,891,121	ENSMUSG00000021823	Q64727

Supplemental Table 3. Shared significantly enriched Gene Ontology Biological Process terms between mouse kidney RNA-seq and human GWAS (MAGMA) analyses.

		Mouse RNA-seq				Human MAGMA				
**GO ID**	**TERM**	**Z**	***P***	**FDR**	**Differential Expression Analysis Significant Genes**	**BETA**	**SE**	***P***	**FDR**	**MAGMA Gene Enrichment Analysis Significant Genes**
GO:0002	regulation of peptide secretion	4.2	1.0×10^{-4}	0.0019	*Cd74, Fgg, Fga, Fgb, Fbn1, Birc5, Lrp1, Oxct1, Cckar, Clcf1, Myh9, Sox4, Acsl4, Aacs, Per2, Stim1, Plcb1, Rph3al, Anxa5, Syt7, Abat, Hmgn3, Sfrp1, Srebf1, Pfkm, Mpc2, Hadh, Slc25a22, Nr1d1, Cpt1a, Camk2n1*	0.20	0.058	2.9×10^{-4}	0.043	*CASR, TNF, HLA-DRB1, TFAP2B, GLUD1*
GO:0042	amide transport	3.7	4.2×10^{-4}	0.0060	*Cd74, Fgg, Fga, Fgb, Cpe, Fbn1, Birc5, Mgst1, Lrp1, Oxct1, Hmga1, Cckar, Clcf1, Myh9, Sox4, Acsl4, Aacs, Lrp2, Slc12a1, Per2, Stim1, Plcb1, Rph3al, Anxa5, Syt7, Myo5a, Abat, Hmgn3, Sfrp1, Srebf1, Pfkm, Slc22a8, Mfsd1, Mpc2, Edn1, Hadh, Slc25a22, Nr1d1, Slc19a1, Cpt1a, Camk2n1*	0.19	0.046	1.9×10^{-5}	0.015	*CASR, ABCG2, TNF, HLA-DRB1, TFAP2B, GLUD1, UMOD, SLC14A1*
GO:0001	ossification	3.2	1.5×10^{-3}	0.016	*Ccn1, Mgp, Cbs, Spp1, Stc1, Kl, Gpc3, Ccn2, Col1a2, Sox9, Col1a1, Fosl2, Fhl2, Slc8a1, Cebpd, Sbno2, Id3, Mmp14, Junb, Id1, Rsad2, Col5a2, Ebp, Csf1, Mdk, Col6a1, Wnt7b, Ranbp3l, Csf1r, Ecm1, Dhrs3, Fermt2, Id2, Rogdi, Thra, Npnt, Ifitm1, Sfrp1, Runx1, Foxc1, Cebpb, Plxnb1*	0.16	0.041	4.4×10^{-5}	0.021	*ALPL, MEF2D, PTGS2, WNT5A, CASR, SLC34A1, TNF, CLIC1, INPPL1, MAP2K6*
GO:0048	tissue morphogenesis	3.0	2.7×10^{-3}	0.025	*Ccn1, Lcn2, Mgp, Lrg1, Cd44, Stc1, Gpc3, Aldh1a2, Ankrd1, Sox9, Col4a1, Hes1, Cyp7b1, Egf, Tacstd2, Tgfb1i1, Col3a1, Socs3, Pdgfra, Mmp14, Vasp, Tpm1, Flna, Hbegf, Csf1, Septin4, Col5a1, Pdgfb, Fat1, Mdk, Lrp2, Hs2st1, Gcnt1, Rock2, Tnc, Aldh1a1, Ajuba, Kdm2a, Map3k1, Wnt7b, Gata3, Dag1, Txnrd1, Klf4, Fermt2, Fgf1, Timeless, Npnt, Rhob, Acta2, Kdr, Sfrp1, Ccdc39, Itga3, St14, Nrg1, Runx1, Spry1, Vcl, Foxc1, Plet1, Prox1, Lama5, Mylk, Edn1*	0.12	0.035	1.9×10^{-4}	0.038	*TAL1, WNT5A, CASR, WDR1, PKD2, TNF, CSNK2B, FKBPL, NOTCH4, FOXA1, ZFPM1, MED1, WNT9B, TBX2*

Supplemental Table 4. Summary of publicly available datasets used in this study.

Data type	Study / Cohort	Description	Source	Accession / URL
GWAS summary statistics	BioBank Japan	Japanese urolithiasis GWAS	BBJ PheWeb	https://pheweb.jp/
GWAS summary statistics	UK Biobank + FinnGen	European urolithiasis GWAS	BBJ PheWeb<br>UK Biobank<br>FINNGEN	https://pheweb.jp/<br>https://pan.ukbb.broadinstitute.org/<br>https://www.finngen.fi/en
GWAS summary statistics	Meta-analysis (BBJ + EUR)	Combined urolithiasis GWAS	BBJ PheWeb	https://pheweb.jp/
Single-cell RNA-seq	Miao Z, *et al.*	Mouse whole-kidney scRNA-seq	Gene Expression Omnibus	https://www.ncbi.nlm.nih.gov/geo/query/acc.cgi?acc=GSE157079
Single-cell RNA-seq	KPMP consortium	Human kidney biopsy scRNA-seq	KPMP consortium	https://www.kpmp.org/
Bulk microarray	Taguchi K, *et al.*	Human reanal papillae bulk microarray	Gene Expression Omnibus	https://www.ncbi.nlm.nih.gov/geo/query/acc.cgi?acc=GSE73680
eQTL	NephQTL	Human kidney eQTL (tubule & glomerulus)	NephQTL	https://nephqtl.org/
